# Regulation of indole‐3‐acetic acid biosynthesis and consequences of auxin production deficiency in *Serratia plymuthica*

**DOI:** 10.1111/1751-7915.14296

**Published:** 2023-06-22

**Authors:** Miriam Rico‐Jiménez, Salvador Muñoz‐Mira, Cristina Lomas‐Martínez, Tino Krell, Miguel A. Matilla

**Affiliations:** ^1^ Department of Biotechnology and Environmental Protection, Estación Experimental del Zaidín Consejo Superior de Investigaciones Científicas Granada Spain

## Abstract

Indole‐3‐acetic acid (IAA) is emerging as a key intra‐ and inter‐kingdom signal molecule that modulates a wide range of processes of importance during plant–microorganism interaction. However, the mechanisms by which IAA carries out its functions in bacteria as well as the regulatory processes by which bacteria modulate auxin production are largely unknown. Here, we found that IAA synthesis deficiency results in important global transcriptional changes in the broad‐range antibiotic‐producing rhizobacterium *Serratia plymuthica* A153. Most pronounced transcriptional changes were observed in various gene clusters for aromatic acid metabolism, including auxin catabolism. To delve into the corresponding molecular mechanisms, different regulatory proteins were biochemically characterized. Among them, a TyrR orthologue was essential for IAA production through the activation of the *ipdc* gene encoding a key enzyme for IAA biosynthesis. We showed that TyrR specifically recognizes different aromatic amino acids which, in turn, alters the interactions of TyrR with the *ipdc* promoter. Screening of mutants defective in various transcriptional and post‐transcriptional regulators allowed the identification of additional regulators of IAA production, including PigP and quorum sensing‐related genes. Advancing our knowledge on the mechanisms that control the IAA biosynthesis in beneficial phytobacteria is of biotechnological interest for improving agricultural productivity and sustainable agricultural development.

## Graphical abstract

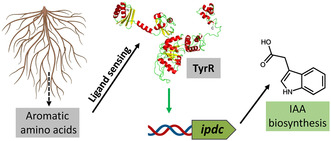

## INTRODUCTION

Indole‐3‐acetic acid (IAA) is the most abundant naturally occurring auxin phytohormone and plays a critical role in plant growth, development and responses against a broad range of biotic and abiotic stresses (Martin‐Arevalillo & Vernoux, 2023; Waadt et al., 2022; Zhao, 2018). Remarkably, auxin biosynthesis is not restricted to plants, but is an ubiquitous signal molecule found in all kingdoms of life (Duca & Glick, 2020), controlling processes as diverse as inflammatory and carcinogenic processes in humans (Addi et al., 2019; Tintelnot et al., 2023), microalgal growth (Lin et al., 2022) or fungal physiology and sporulation (Nicastro et al., 2021).

Analogously to plants (Casanova‐Sáez et al., 2021), bacteria possess several pathways for IAA biosynthesis, with the aromatic amino acid L‐tryptophan being the main precursor (Duca & Glick, 2020). IAA production and secretion are widely distributed in bacteria that establish interactions with plants, both in beneficial and phytopathogenic microbes (Duca & Glick, 2020) – a feature that reflects the importance of IAA in the interaction between plants and bacteria (Duca & Glick, 2020; Eichmann et al., 2021; Kunkel & Johnson, 2021; Spaepen & Vanderleyden, 2011). Thus, IAA production by beneficial microbes promotes plant growth and defence against phytopathogens (Duca et al., 2014; Eichmann et al., 2021; Stringlis et al., 2018). Additionally, IAA produced by various phytopathogenic bacteria stimulates virulence through the alteration of auxin homeostasis in the plant or the suppression of the plant's defensive responses (Eichmann et al., 2021; Kunkel & Johnson, 2021).

In addition to its role as an inter‐kingdom signal molecule, there is mounting evidence for the role of IAA as a bacterial signalling molecule, including the regulation of metabolic and physiological processes that are important for establishing successful interactions with plants. Among these processes, IAA was shown to modulate biofilm formation (Donati et al., 2013; Imperlini et al., 2009), stress resistance (Bianco & Defez, 2009; Imperlini et al., 2009), antibiotics biosynthesis (Gavira et al., 2023; Matilla et al., 2018; Wang et al., 2016), production of virulence factors (Kunkel & Johnson, 2021), chemotaxis (Rico‐Jiménez et al., 2022), catabolism (Conway et al., 2022) and plant colonization (Duca & Glick, 2020; Kunkel & Johnson, 2021). To investigate the mechanisms of these processes, several transcriptomic analyses have previously been conducted in different bacteria exposed to exogenous IAA or using mutants deficient in IAA synthesis. These bacteria included the phytopathogens *Agrobacterium tumefaciens* (Yuan et al., 2008) and *Pseudomonas syringae* (Djami‐Tchatchou et al., 2022), as well as the nitrogen‐fixing bacteria *Bradyrhizobium japonicum* (Donati et al., 2013), *Azospirillum brasilense* (Van Puyvelde et al., 2011) and *Sinorhizobium meliloti* (Defez et al., 2016; Imperlini et al., 2009). However, to the best of our knowledge, there are no global transcriptional data available on non‐symbiotic phytobacteria to elucidate the mechanisms by which these microbes sense and respond to IAA.

Given the importance of IAA in bacterial physiology and metabolism, as well as in the interaction between bacteria and their hosts, its biosynthesis can be expected to be tightly regulated. In fact, bacteria present several mechanisms to control auxin levels, which not only include the control of expression of the corresponding genes and proteins but also involve reducing the levels of the free form of IAA (the biologically active form) through its conjugation and degradation (Duca & Glick, 2020). In this context, the pre‐ and post‐transcriptional regulatory mechanisms that modulate bacterial IAA biosynthesis are poorly understood. In fact, only a few regulators have been reported to be involved in the control of IAA production. Among them, the TyrR regulator in *Enterobacter ludwigii* (Coulson et al., 2020; Ryu & Patten, 2008), the sigma factor RpoS in *Pseudomonas* spp. (Oh et al., 2013; Patten & Glick, 2002b) and the GacS/GacA system in *Pseudomonas chlororaphis* (Kang et al., 2006). Several regulators of the AraC and LysR families were hypothesized to be involved in the regulation of IAA biosynthesis (Duca & Glick, 2020), but their role in this process remains to be investigated.

The wheat rhizosphere isolate *Serratia plymuthica* A153 is a broad‐spectrum antibiotic producer and a model bacterium for studying biosynthesis and regulation of new bioactive molecules potentially useful for biocontrol of agricultural diseases. A153 devotes ~5% of its genome to the synthesis of secondary metabolites (Matilla, Drew, et al., 2016), including the antibacterial andrimid (Matilla, Nogellova, et al., 2016), the antifungals oocydin A (Matilla et al., 2015) and pyrrolnitrin (De Vleesschauwer & Hofte, 2007), as well as the nematicide zeamine (Hellberg et al., 2015). Notably, our previous research showed that IAA produced by phytobacterial competitors regulates the production of the antibiotic andrimid in *S. plymuthica* A153 (Matilla et al., 2018). We also established, for the first time, the molecular mechanism by which IAA controls antibiotic production, which is based on the specific recognition of auxins by the transcriptional regulator AdmX (Gavira et al., 2023; Matilla et al., 2018). However, although we showed that A153 efficiently synthesizes IAA from L‐tryptophan through the indole‐3‐pyruvate (IPA) pathway, endogenous IAA does not play a role in the regulation of antibiotics production in A153 (Matilla et al., 2018). Given that *S. plymuthica* A153 cannot use IAA as nutrient source, this rhizobacterium serves as an excellent model to investigate the physiological role of endogenous IAA as a signal in beneficial plant‐associated bacteria.

In this study, we took advantage of the identification of the enzyme indole‐3‐pyruvate decarboxylase (IPDC) in *S. plymuthica* A153 (Matilla et al., 2018), responsible for converting IPA to indole‐3‐acetaldehyde during IAA biosynthesis (Duca & Glick, 2020), to investigate the role of IAA as an endogenous bacterial signal molecule. We found that *ipdc* deletion causes a severe reduction in IAA levels as well as major changes in the A153 transcriptome. Using multidisciplinary approaches, we identified and characterized different regulators involved in modulating IAA production in this important biocontrol agent.

## EXPERIMENTAL PROCEDURES

### Strains, plasmids, oligonucleotides and culture conditions

Bacteria and fungi used in this study are described in Table S1. Plasmids and oligonucleotides are listed in Tables S2 and S3, respectively. *Serratia plymuthica* strains were grown routinely at 30°C, unless otherwise indicated, in lysogeny broth (LB; 5 g/L yeast extract, 10 g/L bacto tryptone and 5 g/L NaCl), potato dextrose (PD; 16 g/L; Difco) or minimal medium (0.1% (w/v) (NH_4_)_2_SO_4_, 0.41 mM MgSO_4_, 40 mM K_2_HPO_4_, 14.7 mM KH_2_PO_4_, pH 6.9–7.1) with 15 mM glucose as carbon source, unless otherwise indicated. *Escherichia coli* strains were grown at 37°C in LB. *E. coli* DH5α was used as a host for gene cloning. Media for propagation of *E. coli* β2163 were supplemented with 300 μM 2,6‐diaminopimelic acid. When appropriate, antibiotics were used at the following final concentrations (in μg/mL): ampicillin, 100; kanamycin, 50; streptomycin, 50; tetracycline, 10 (*E. coli* strains) and 15 (*S. plymuthica* strains); gentamicin, 10 (*E. coli*) and 25 (*S. plymuthica*). Sucrose was added to a final concentration of 10% (w/v) when required to select derivatives that had undergone a second crossover event during marker‐exchange mutagenesis.

### In vitro nucleic acid techniques

Plasmid DNA was isolated using the NZY‐Miniprep kit (NZY‐Tech). For DNA digestion, alkaline phosphatase and ligation reactions, manufacturers' instructions were followed (New England Biolabs and Roche). DNA fragments were recovered from agarose gels using the Qiagen gel extraction kit. PCR reactions were purified using the Qiagen PCR Clean‐up kit. PCR fragments were verified by DNA sequencing carried out at the Institute of Parasitology and Biomedicine Lopez‐Neyra (CSIC; Granada, Spain). The Mix & Go transformation kit (Zymo Research, Cat. No.: T3002) was used to prepare *E. coli* competent cells, and transformations were performed using standard protocols (Sambrook et al., 1989). Phusion^®^ high‐fidelity DNA polymerase (Thermo Fisher Scientific) was used for the amplification of PCR fragments.

### Construction of bacterial mutant strains and complementation plasmids

Chromosomal mutants defective in *ipdc*, *tyrR*, *AWY96_RS13985*, *AWY96_RS19325*, *AWY96_RS21200* and *pigP* were constructed by homologous recombination using derivate plasmids of pKNG101. These plasmids are listed in Table S2 and were generated by amplifying a 0.6–0.9 kb flanking regions of the gene to be mutated using primers listed in Table S3. The resulting PCR products were digested with the enzymes specified in Table S2 and ligated in a three‐way ligation into pUC18Not, previously to be cloned into the marker exchange vector pKNG101. In all cases, plasmids for mutagenesis were transferred to *S. plymuthica* by biparental conjugation using *E. coli* β2163. For the construction of the plasmid for complementation assays, the *ipdc* and *tyrR* genes were amplified using primers listed in Table S3 and cloned into pBBR‐based plasmids. The resulting plasmids were transformed into *S. plymuthica* strains by electroporation. All plasmids and mutations were verified by PCR and sequencing.

### β‐Galactosidase assays

Expression of the *lacZ* reporter gene was monitored during growth using 2‐nitrophenyl β‐D‐galactopyranoside (ONPG; Merck) as a substrate, as described previously (Miller, 1972). The transcriptional fusion assays were carried out using *S. plymuthica* A153 LacZ (control) or derived mutants.

### Growth experiments

A153 strains were grown overnight in a minimal medium containing either 5 mM phenylacetic acid (PAA), 5 mM 4‐hydroxybenzoate (4HBA) or 5 mM 4‐hydroxyphenylacetic acid (4HPA) as a sole carbon source. Cultures were washed twice with M9 salts (7 g/L Na_2_HPO_4_ × 7H_2_O, 3 g/L KH_2_PO_4_, 0.5 g/L NaCl, 1 g/L NH_4_Cl; pH, 7.0) and then diluted to an OD_600_ of 0.02 in either minimal medium supplemented with 5 mM PAA, 5 mM 4HBA or 5 mM 4HPA as a sole carbon source. Then, 200 μL of these cultures were transferred into microwell plates and growth at 30°C was followed on a Bioscreen microbiological growth analyser for 96 h (Oy Growth Curves Ab Ltd., Helsinki, Finland).

### Swimming motility assays

Overnight cultures were adjusted to an OD_600_ of 1 and 3 μL of these cultures were spotted onto LB‐Difco agar (0.3% [w/v]) plates and incubated at 30°C for 24 h.

### Quantification of IAA by the Salkowski assay

Bacterial strains were adjusted to an OD_600_ of 0.075 in LB medium and grown at 30°C in the presence of 1 mg/mL of L‐Trp. After 10 and 24 h, culture samples were pelleted by centrifugation (7000 × *g* for 10 min) and the supernatants were filtered (0.2 μM cut‐off). One millilitre of the resulting supernatants was mixed with 2 mL of Salkowski's reagent (Patten & Glick, 2002a) and incubated at room temperature for 30 min before measuring the OD_535_. IAA concentrations were inferred from a standard curve obtained with commercial IAA (Merck).

### Quantification of IAA by gas chromatography–mass spectrometry (GC–MS)

Triplicate 20 mL cultures of each bacterial strain were adjusted to an OD_600_ of 0.075 in LB medium and grown at 30°C in the presence of 1 mg/mL of L‐Trp. After 10 and 24 h, culture samples were pelleted by centrifugation (7000 × *g* for 10 min) and the supernatants were filtered (0.2 μM cut‐off). Subsequently, 5 μg/mL of 5‐methoxy‐indole‐3‐acetic acid (5‐Me‐IAA; Merck) was added as internal standard to the filtered supernatants and IAA was extracted as described previously (Gutierrez et al., 2009), with minor modifications. Briefly, the pH of 3 mL filter‐sterilized supernatants was lowered to pH 2.5–3.0 by the addition of HCl and each supernatant was extracted twice with 2.5 mL of diethyl ether (analytically pure). Ether phases were combined and evaporated to dryness under a stream of N_2_ gas. Samples were subsequently reconstituted in 100 μL of BSTFA + TMCS (N,O‐bis(trimethylsilyl)triluoroacetamide and trimethylchlorosilane (99:1, Merck)) and trimethylsilyated for 1 h at 70°C. After cooling, 1 μL was analysed with a Varian 450 gas chromatograph‐240 ion trap mass spectrometer. Electron impact ionization at 70 eV was used. GC–MS conditions were as follows: column, DB‐5 (30 m by 0.25 mm by 0.025‐mm film thickness); carrier gas, He at 1 mL/min; injection temperature, 280°C; initial temperature, 60°C for 5 min, increasing by 11°C/min to a final temperature of 300°C; using a detector temperature of 290°C. Mass‐to‐charge ratios (m/z values) from 45 to 600 were monitored using the TIC Full Scan mode to obtain the chromatograms. Integrated IAA and 5‐Me‐IAA peak areas (in SIM mode for 130 and 160 m/z respectively) were compared to standard curves for authentic IAA and 5‐Me‐IAA and used to calculate the IAA concentration.

### Protein overexpression and purification

*Escherichia coli* BL21(DE3) harbouring plasmids pET28b‐tyrR, pET28b‐hpaA and pET28b‐hpaR were grown in 2 L Erlenmeyer flasks containing 500 mL LB medium supplemented with kanamycin. Cultures were grown under continuous stirring (200 rpm) at 30°C. In all cases, at an OD_600_ of 0.5, protein expression was induced by the addition of isopropyl‐β‐D‐thiogalactopyranoside (IPTG) to a final concentration of 0.25 mM and growth was continued overnight (approximately 18 h) at 18°C. Cells were pelleted by centrifugation at 20,000 × *g* for 20 min. Proteins were purified by metal affinity chromatography. Briefly, the cell pellets of HpaA_A153_ and HpaR_A153_ were resuspended in buffer A (20 mM Tris, 300 mM NaCl, 2 mM EDTA, 10% (v/v) glycerol, 5 mM β‐mercaptoethanol, 10 mM imidazole; pH 8.0), whereas TyrR_A153_ was resuspended in buffer B (20 mM Tris, 500 mM NaCl, 2 mM EDTA, 5% (v/v) glycerol, 5 mM β‐mercaptoethanol, 10 mM imidazole; pH 8) containing cOmplete™ protease inhibitor (Roche) and Benzonase (Merck). Cells were then broken by French press treatment at a gauge pressure of 62.5 lb/in^2^. After any remaining cellular debris was pelleted by centrifugation at 10,000 × *g* for 1 h, the supernatant was loaded onto a 5 mL HisTrap column (Amersham Bioscience) equilibrated with buffers A or B. Proteins were eluted by a gradient of 40–500 mM imidazole in the same buffers.

### Isothermal titration calorimetry (ITC) assays

Measurements were made using a VP‐ITC microcalorimeter (MicroCal, Inc., Northampton, MA) at 10–25°C. HpaA_A153_ and HpaR_A153_ were dialyzed into 50 mM Tris, 300 mM NaCl, 10% (v/v) glycerol, 2 mM β‐mercaptoethanol, pH 8.0. Alternatively, TyrR_A153_ was dialyzed into 5 mM Tris, 5 mM PIPES, 5 mM MES, 150 mM NaCl, 10 mM MgCl_2_, 10 mM β‐mercaptoethanol, 10% (v/v) glycerol, pH 7.5. Proteins at 5–50 μM were placed into the sample cell and titrated with 3.2–12.8 μL aliquots of 0.5–10 mM ligand solutions made up in the corresponding dialysis buffers. For DNA binding studies, wild‐type and mutant oligonucleotides corresponding to the TyrR box were synthesized and annealed. Briefly, an equimolar mixture of 200 μM of each complementary oligonucleotide was prepared in 10 mM Tris, 50 mM NaCl, 1 mM EDTA, pH 7.5. This mixture was subsequently incubated at 95°C for 5 min, chilled on ice and dialyzed in the same buffer used for TyrR_A153_ ITC studies. A typical experiment consisted in an injection of 3.2–12.8 μL aliquots of 100 μM DNA into a 5–20 μM protein solution in the absence and presence of TyrR ligands (e.g. 1 mM L‐Trp; 1 mM L‐Phe; 0.1 mM L‐Tyr; 0.5 mM ATP). Protein‐DNA interaction studies were done at 10°C. In all cases, the mean enthalpies measured from the injection of the ligand in the buffer were subtracted from raw titration data before data analysis with the ORIGIN software (MicroCal).

### Antibacterial and antifungal assays

Antagonistic activities against bacteria and plant‐pathogenic fungi and oomycetes were assayed as described previously (Matilla et al., 2012). Antibacterial and antifungal assays were conducted in LB and PD agar medium, respectively, at 25°C for 24 h (antibacterial assays) and 96 h (antifungal assays).

### Competitive root colonization assays

Sterilization, germination and inoculation of maize seeds were carried out as described previously (Matilla et al., 2007). Briefly, sterile seeds were incubated at 30°C for 45 min with a 10^7^ CFU/mL 1:1 mixture of A153 and Δ*ipdc*‐km or Δ*tyrR*‐km. Thereafter, seeds were rinsed with sterile deionized water and planted in 50 mL Sterilin tubes containing 40 g of sterile washed silica sand and 10% (v/w) plant nutrient solution supplemented with Fe‐EDTA and micronutrients. Plants were maintained at 24°C with a daily light period of 16 h. After 7 days, bacterial cells were recovered from the rhizosphere, as described previously (Matilla et al., 2007). Serial dilutions were plated on LB‐agar and LB‐agar medium supplemented with 50 μg/mL of kanamycin to select the *ipdc* or *tyrR* mutant strains.

### RNA extraction, cDNA synthesis and quantitative real‐time PCR (RT‐qPCR) analyses

Total RNA was extracted by using TRI Reagent^®^ (Invitrogen) followed by Turbo DNase treatment (Ambion) and RNA clean‐up with RNeasy Mini Kit (Qiagen), according to manufacturers´ instructions. The RNA concentration was determined spectrophotometrically, and RNA degradation and contamination were assessed by electrophoresis on 2% (w/v) agarose gels. The synthesis of cDNA was performed using random hexamers (GE Healthcare) and SuperScript II reverse transcriptase (Invitrogen) in a 25 μL reaction volume with 1 μg of total RNA and incubation at 42°C for 2 h. RT‐qPCRs were performed as described previously (Matilla, Nogellova, et al., 2016) using primers described in Table S3. RT‐qPCR amplifications were performed using the iQ™ SYBR^®^ Green supermix (Bio‐Rad) in an MyiQ™ 2 Two‐Colour Real‐Time PCR Detection System (Bio‐Rad) associated with iQ5 optical system software (version 2.1.97.1001). To confirm the absence of contaminating genomic DNA, control PCRs were carried out using no‐RT cDNA samples as templates. Melting curve analyses were conducted to ensure the amplification of a single product. The relative gene expression was calculated using the critical threshold (ΔΔCt) method (Pfaffl, 2001) using the *gyrB* gene as reference for data normalization.

### RNA‐seq and data analysis

Before sequencing, RNA purity was checked using the NanoPhotometer^®^ spectrophotometer (IMPLEN, CA, USA). RNA integrity and quantification were assessed using the RNA Nano 6000 Assay Kit of the Bioanalyzer 2100 system (Agilent Technologies, CA, USA). One microgram of total RNA per sample (two conditions, three biological replicates per condition) was used to construct the sequencing libraries. These libraries were generated by Novogen (United Kindom) Company Limited using NEBNext^®^ Ultra™ Directional RNA Library Prep Kit for Illumina^®^ (New England Biolabs; #E7530), following the manufacturer's recommendations. Prior to library construction, ribosomal RNA was depleted with Illumina Ribo‐Zero Plus rRNA Depletion Kit (Illumina; Ref. 20,037,135), according to the manufacturer's instructions. The resulting libraries were quantified by quantitative PCR and the inserts measured using a LabChip GX instrument and the LabChip NGS 3 K reagent kit (PerkinElmer; CLS960013). Libraries were sequenced using the NovaSeq 6000 Illumina platform and paired‐end reads were generated. Raw data of FASTQ format were first processed by Novogene in‐house scripts. In this step, clean reads were obtained by removing any containing adapter sequences or where uncertain nucleotides represented more than 10% of the read length. Reads with low‐quality nucleotides (base quality <20) in more than 50% of the read length were also discarded. Paired‐end clean reads were mapped to the reference genome (GenBank under the accession number LRQU00000000) using Bowtie2 software v.2.3.4.3. FeatureCounts software was used to count the reads mapped to each gene, including known and novel genes. Differential expression analysis between conditions was performed using DESeq2 package v1.20.0 in R. The resulting *p*‐values were adjusted using the Benjamini and Hochberg's approach for controlling the false discovery rate. Genes with an adjusted *p*‐value (*p*_adj_) < 0.05 found by DESeq2 were assigned as differentially expressed.

## RESULTS

### Expression of the *ipdc* gene in *S. plymuthica* A153 occurs in the stationary growth phase

**FIGURE 1:**
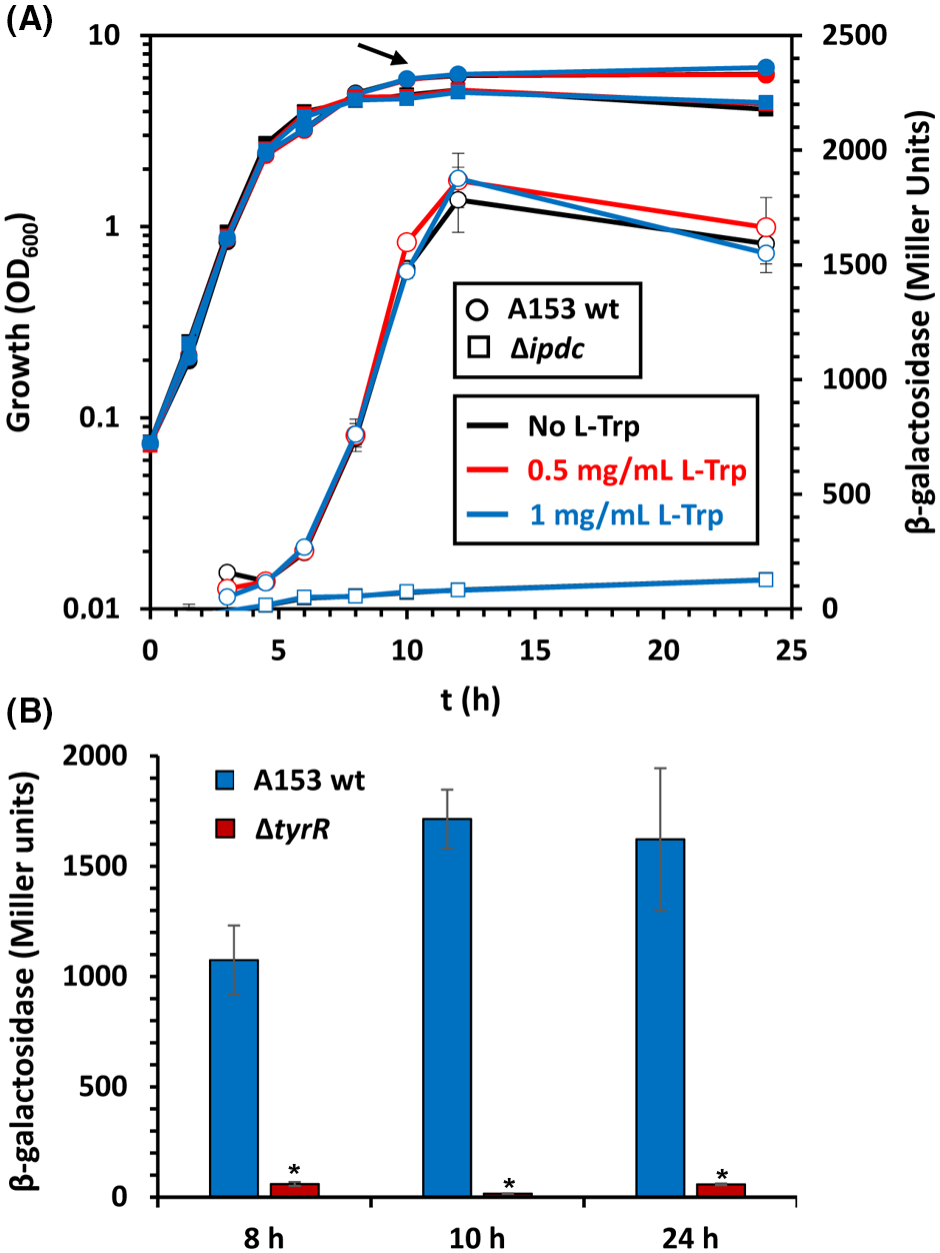
The expression of the *ipdc* gene of *Serratia plymuthica* A153 is growth phase‐dependent and controlled by TyrR. (A) Transcription of the *ipdc* (P_*ipdc*_ *::lacZ*; pMAMV302) promoter fusion throughout growth of *S. plymuthica* A153. β‐galactosidase activity (open symbols) and growth curves (filled symbols) were determined in LB medium at 30°C in the absence and presence of different concentrations of L‐Tryptophan (L‐Trp). Data are the mean and standard deviation of three biological replicates. Arrow, time point when samples for RNA‐seq were taken. Wt, wild‐type; OD_600_, optical density at 600 nm. (B) Transcription of the *ipdc* (P_*ipdc*_ *::lacZ*; pMAMV302) promoter fusion throughout growth in LB medium supplemented with 1 mg/mL L‐Trp at 30°C in different *S. plymuthica* A153 strains. *p < 0.01, Student's t‐test of *tyrR* mutant with respect to the A153 wild‐type (wt) strain. No growth defect was observed for the *tyrR* deficient strain under these conditions.

To investigate the expression profile of the *ipdc* (*AWY96_RS14025*) gene in A153, we constructed a transcriptional fusion P_*ipdc*_::*lacZ* in plasmid pMAMV302 and its β‐galactosidase activity was assessed in LB medium throughout growth. Transcription from the *ipdc* promoter started in the early stationary phase of growth and reached an apparent maximum in the late stationary phase (Figure 1A). Supplementation of LB medium with different concentrations of L‐Trp, the precursor of IAA in strain A153, did not result in increased *ipdc* transcription (Figure 1A).

**FIGURE 2:**
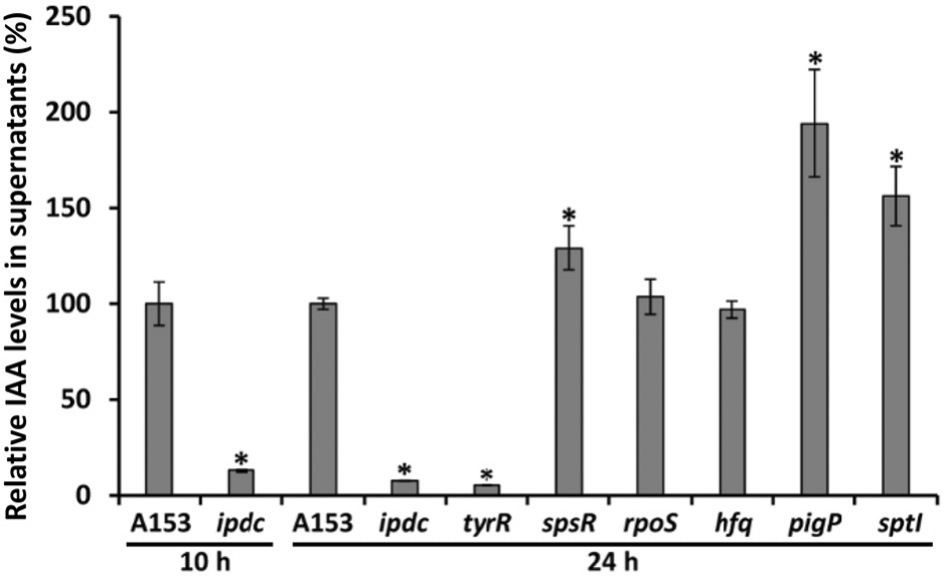
Quantification of indole‐3‐acetic acid (IAA) production by *Serratia plymuthica* A153 strains measured by gas chromatography coupled to mass spectrometry. Shown are the relative IAA levels in supernatants of different A153 strains grown at 30°C for 10 and 24 h in LB supplemented with 1 mg/mL L‐Trp. Means and standard deviations of three biological replicates are shown. *p < 0.01, Student's *t*‐test of mutant strains with respect to the A153 wild‐type strain.

We subsequently analysed the supernatants of the parental strain A153 and an *ipdc* deficient mutant by gas chromatography coupled to mass spectrometry (GC–MS) to determine IAA production levels in both strains. The results showed that IAA levels of the *ipdc* mutant were largely reduced, namely by 87.9% and 92.5%, with respect to the wild‐type strain after 10 and 24 h, respectively (Figure 2). Given that IAA, the end product of the IPA pathway (Duca & Glick, 2020), was shown to induce expression of *ipdc* in different plant‐associated bacteria by positive feedback (Malhotra & Srivastava, 2008; Vande Broek et al., 2005), we analysed the transcription of the *ipdc* gene in a *ipdc* mutant background. β‐Galactosidase assays revealed that *ipdc* deletion abolished transcription from the *ipdc* promoter (Figure 1A). To determine whether exogenous supplementation of IAA could restore *ipdc* expression, β‐galactosidase activity was assessed in wild‐type A153 and the *ipdc* mutant in the absence and presence of different IAA concentrations, namely 0.25 mM and 1 mM IAA. We found that IAA supplementation did not result in altered *ipdc* expression in any of the genetic backgrounds analysed (Figure S1), suggesting that an alternative metabolite derived from the activity of the IPDC enzyme may be responsible for the modulation of *ipdc* expression.

### Deletion of *ipdc* causes important changes in A153 transcript levels

The ability of IAA to modulate gene expression, together with the fact that it is synthesized in non‐host environments (Duca & Glick, 2020; Kunkel & Johnson, 2021; Matilla et al., 2018) and that it is a lipophilic acid that can diffuse across cell membranes (pK_a_ = 4.8) (Patten et al., 2013), has suggested that this auxin could act as a quorum sensing‐like molecule (Duca & Glick, 2020). Given the very distinct IAA production levels in A153 and its *ipdc* mutant (Figure 2), we investigated the role of the endogenous IAA as a signal molecule by comparing the global transcriptome of the parental strain A153 to that of the *ipdc* mutant. For this purpose, because *ipdc* expression was maximal in stationary phase (Figure 1A), RNA was prepared from cultures grown in LB supplemented with 1 mg/mL L‐Trp at early stationary phase of growth. Under these conditions, the IAA concentration determined by quantitative GC–MS in the supernatants of the wild‐type A153 and its *ipdc* deficient strain were 9.4 ± 0.8 μM and 1.2 ± 0.1 μM, respectively.

**FIGURE 3:**
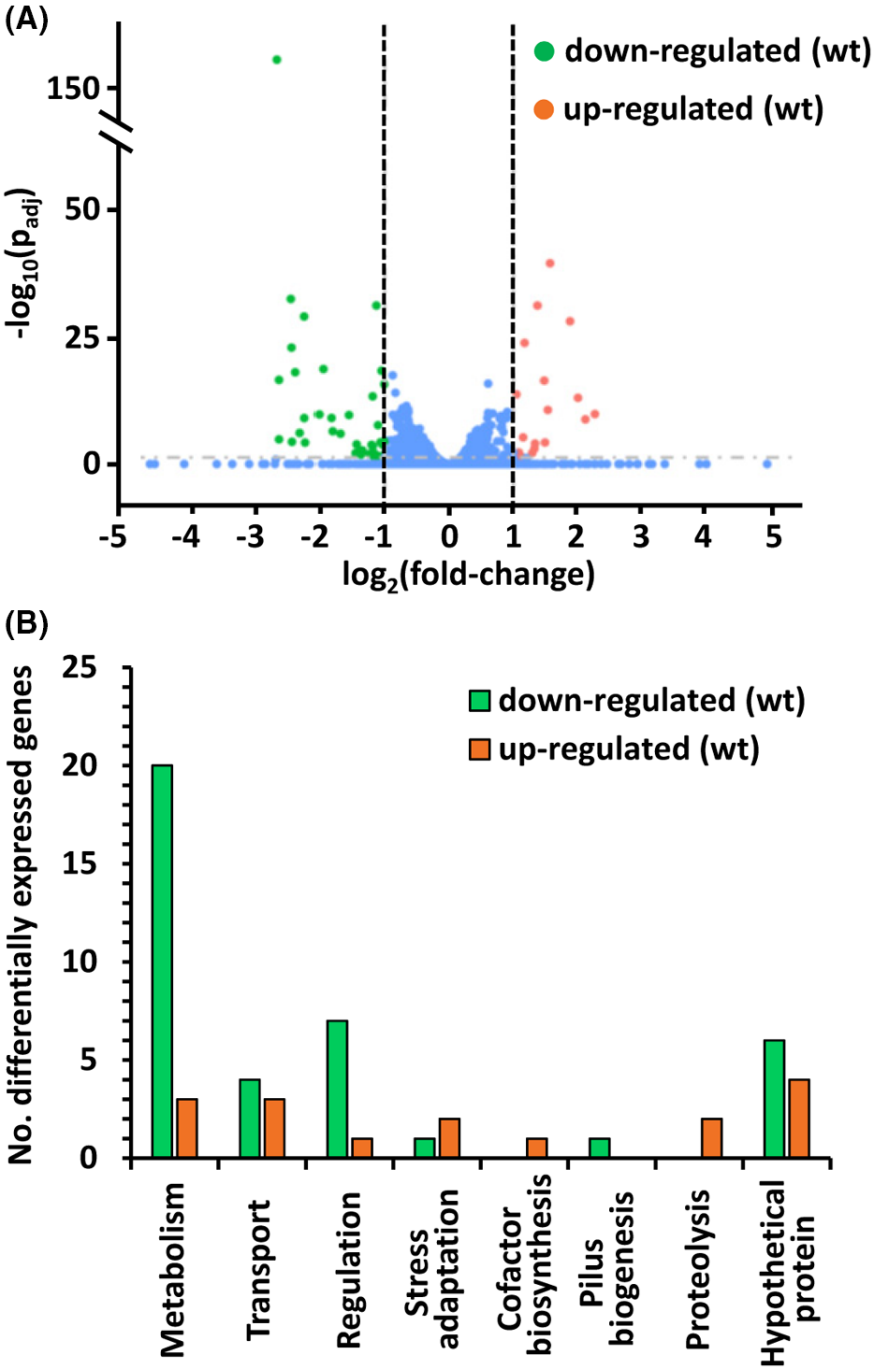
RNA‐seq analysis of wild‐type and *ipdc* mutant of *Serratia plymuthica* A153. (A) Volcano plot of differentially transcribed genes in the ∆*ipdc* mutant as compared to the wild‐type (wt) strain. The log_2_ (fold change) was plotted against the statistical significance (−log_10_ of the adjusted *p*‐value (*p*_adj_) <0.05) for each gene. Vertical dashed lines represent the log_2_ fold change cut‐off of 1 or −1. Blue dots represent insignificant differentially expressed genes. (B) Functional classification of the differentially regulated genes.

**FIGURE 4:**
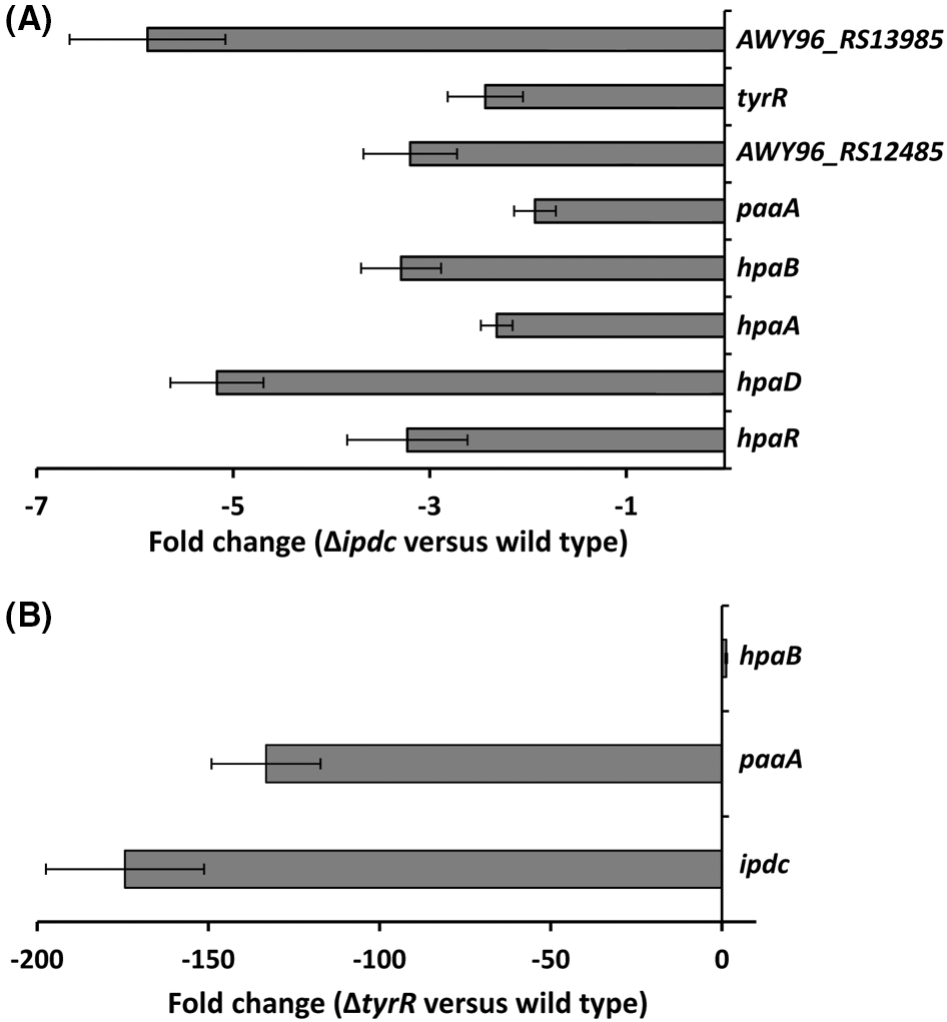
Impact of *ipdc* and *tyrR* deletion on the transcript levels of differentially expressed genes identified in the RNA‐seq analysis. Shown are the fold‐change mRNA levels of selected genes in Δ*ipdc* versus wild‐type (A) and Δ*tyrR* versus wild‐type (B) measured by quantitative RT‐PCR under the same conditions used for the RNA‐seq analysis. Data are the mean and standard error of three biological replicates, each conducted in triplicate.

Statistically differentially expressed genes (DEGs) were defined by a fold‐change magnitude above 2 and an adjusted *p*‐value (*p*_adj_) using the Benjamini and Hochberg's approach inferior to 0.05. A total of 45 DEGs, ~1% of the total genes in the A153 genome, were identified; 16 up‐regulated and 39 down‐regulated in the *ipdc* mutant (Figure 3; Table 1). These DEGs were classified into different functional categories, including metabolism (51%), transport (15%), regulation (18%), stress adaptation (7%) and proteolysis (4%), among others. Twenty‐two percent of the DEGs were of unknown function (Figure 3; Table 1). When possible, DEGs were also classified according to the KEGG Orthology (KO terms). Major down‐regulated processes in the *ipdc* mutant involve amino acid metabolism, xenobiotics biodegradation and metabolism, signalling and cellular processes as well as environmental and genetic information processing. Alternatively, the main up‐regulated processes include metabolism and signalling and cellular processes (Table 1). To validate these RNA‐seq results, transcript levels of a selection of various DEGs, belonging to different functional categories, were analysed by RT‐qPCR. A strong correlation between RNA‐seq and RT‐qPCR was found (Figure 4A).

**TABLE 1:** Differentially expressed *Serratia plymuthica* A153 genes in Δ*ipdc* versus wild‐type.

Locus no.	Gene name	Known or predicted function	Fold change^a^	KEGG Orthology
Downregulated				
Metabolism				
AWY96_RS04335	—	SDR family oxidoreductase	−2.15	LM
AWY96_RS12475	—	Class II histone deacetylase	−2.54	Unclassified
AWY96_RS12500	—	SDR family oxidoreductase	−2.71	Unclassified
AWY96_RS12520	*hpaG1*	Fumarylacetoacetate hydrolase family protein	−5.17	AAM
AWY96_RS12525	*hpaG2*	Fumarylacetoacetate hydrolase family protein	−4.04	AAM
AWY96_RS12530	*hpaE*	5‐Carboxymethyl‐2‐hydroxymuconate semialdehyde dehydrogenase	−4.93	AAM
AWY96_RS12535	*hpaD*	3,4‐Dihydroxyphenylacetate 2,3‐dioxygenase	−5.42	Unclassified
AWY96_RS12540	*hpaF*	5‐Carboxymethyl‐2‐hydroxymuconate Delta‐isomerase	−4.69	AAM
AWY96_RS12545	*hpaH*	2‐Oxo‐hepta‐3‐ene‐1,7‐dioic acid hydratase	−5.35	AAM
AWY96_RS12550	*hpaI*	4‐Hydroxy‐2‐oxoheptanedioate aldolase	−5.38	AAM
AWY96_RS12565	*hpaB*	4‐Hydroxyphenylacetate 3‐monooxygenase, oxygenase component	−6.15	AAM
AWY96_RS12570	*hpaC*	4‐Hydroxyphenylacetate 3‐monooxygenase, reductase component	−3.55	AAM
AWY96_RS13795	—	3‐Deoxy‐7‐phosphoheptulonate synthase	−2.25	AAM
AWY96_RS13985	—	Aldehyde dehydrogenase family protein	−6.30	AAM
AWY96_RS14025	*ipdc*	Indolepyruvate decarboxylase	−2.1	Unclassified
AWY96_RS21745	—	4‐Hydroxybenzoate 3‐monooxygenase	−2.28	XBM
AWY96_RS24295	—	3‐Oxoacid CoA‐transferase subunit A	−2.28	XBM
AWY96_RS24300	—	3‐Oxoacid CoA‐transferase subunit B	−2.15	XBM
AWY96_RS24485	*paaA*	1,2‐Phenylacetyl‐CoA epoxidase subunit A	−2.19	AAM
AWY96_RS24495	*paaC*	Phenylacetate‐CoA oxygenase subunit PaaC	−1.55^b^	AAM
AWY96_RS24500	*paaD*	Phenylacetate‐CoA oxygenase subunit PaaJ	−1.56^b^	AAM
AWY96_RS24510	*paaF*	2,3‐Dehydroadipyl‐CoA hydratase	−1.61^b^	AAM
AWY96_RS24515	*paaG*	2‐(1,2‐Epoxy−1,2‐dihydrophenyl)acetyl‐CoA isomerase PaaG	−1.55^b^	AAM
AWY96_RS24525	*paaI*	Hydroxyphenylacetyl‐CoA thioesterase PaaI	−1.55^b^	AAM
AWY96_RS24530	*paaJ*	Phenylacetate‐CoA ligase	‐1.54^b^	AAM
AWY96_RS24715	*idi*	Isopentenyl‐diphosphate delta‐isomerase	−2.07	Unclassified
Transport				
AWY96_RS03305	—	LysE family translocator	−2.30	Unclassified
AWY96_RS07550	*panS*	Ketopantoate/pantoate/pantothenate transporter PanS	−2.58	SCP
AWY96_RS12555	*hpaX*	4‐Hydroxyphenylacetate permease	−4.70	SCP
AWY96_RS21750	*pcaK*	Major facilitator superfamily (MFS) transporter. 4‐Hydroxybenzoate transported. Homology to transporter PcaK	−3.50	SCP
Transcriptional regulators, regulatory proteins and sensor proteins				
AWY96_RS12455	—	LuxR C‐terminal‐related transcriptional regulator	−3.22	Unclassified
AWY96_RS12515	*hpaR*	MarR family homoprotocatechuate degradation operon regulator HpaR	−2.94	Unclassified
AWY96_RS12560	*hpaA*	AraC family 4‐hydroxyphenylacetate catabolism regulatory protein HpaA	−6.15	GIP
AWY96_RS12485	—	LuxR family transcriptional regulator	−3.87	Unclassified
AWY96_RS22350	*tyrR*	Transcriptional regulator TyrR	−2.01	GIP
AWY96_RS24145	*fhlD*	Flagellar transcriptional regulator FlhD	−2.58	EIP
AWY96_RS24535	*paaX*	Phenylacetic acid degradation operon negative regulatory protein PaaX	−1.50^b^	GIP
Stress adaptation, detoxification & antibiotic resistance				
AWY96_RS18365	—	Efflux RND transporter periplasmic adaptor subunit	−2.35	SCP
Pilus biogenesis				
AWY96_RS08510	—	Fimbria/pilus outer membrane usher protein	−2.21	SCP
Unknown function				
AWY96_RS00800	—	Hypothetical protein	−2.23	Unclassified
AWY96_RS11255	—	Hypothetical protein	−2.05	Unclassified
AWY96_RS12465	—	Hypothetical protein	−2.01	Unclassified
AWY96_RS18820	—	Putative recombinase/integrase	−2.74	Unclassified
AWY96_RS21605	—	Hypothetical protein	−4.64	Unclassified
AWY96_RS24415	—	Hypothetical protein	−2.01	Unclassified
Upregulated				
Metabolism				
AWY96_RS04070	*grcA*	Autonomous glycyl radical cofactor GrcA	2.96	Unclassified
AWY96_RS12110	*—*	Transglutaminase family protein	2.18	Unclassified
AWY96_RS17570	*hcp*/*priS*	Hydroxylamine reductase	2.12	EM
Transport				
AWY96_RS12985	—	Threonine/serine exporter ThrE family protein	2.80	Unclassified
AWY96_RS13100		DASS family sodium‐coupled anion symporter	2.78	SCP
AWY96_RS13150	*alaE*	L‐alanine exporter AlaE	2.22	Unclassified
Stress adaptation, detoxification & antibiotic resistance				
AWY96_RS09195	*emrD*	Multidrug efflux MFS transporter EmrD	2.51	SCP
AWY96_RS18945	—	Universal stress protein	2.26	SCP
Proteolysis				
AWY96_RS11905	—	U32 family peptidase	2.45	M
AWY96_RS19140	*pepT*	Peptidase T	4.80	M
Transcriptional regulators, regulatory proteins and sensor proteins				
AWY96_RS01195	*dpiA*	Two‐component response regulator DpiA	2.52	EIP
Biosynthesis of cofactors, prosthetic groups and carriers				
AWY96_RS20480	*bioD*	Dethiobiotin synthase	2.89	MCV
Unknown function				
AWY96_RS01420	—	Hypothetical protein	4.00	Unclassified
AWY96_RS02200	—	YfbU family protein	3.67	Unclassified
AWY96_RS13970	—	Hypothetical protein	2.06	Unclassified
AWY96_RS22160	—	Hypothetical protein	4.33	Unclassified

Abbreviations: AAM, amino acid metabolism; EM, energy metabolism; EIP, environmental information processing; GIP, genetic information processing; LM, lipid metabolism; M, metabolism; MCV, metabolism of cofactors and vitamins; SCP, signalling and cellular processes; XBM, xenobiotics biodegradation and metabolism.

^a^ *p*‐Value adjusted lower than 0.05.

^b^ Genes that were included due to the general down‐regulation of the *paa* phenylacetic acid catabolic operon at least 1.5‐fold.

### An auxin catabolic pathway is repressed in the *ipdc* mutant

RNA‐seq data revealed the down‐regulation in the *ipdc* mutant of the *paa* and *hpa* catabolic genes clusters encoding the pathways responsible for the degradation of the natural auxin phenylacetic acid (PAA) and its hydroxylated derivative 4‐hydroxyphenylacetic acid (4HPA), respectively (Table 1; Figure 4A and Figure S2) (Díaz et al., 2001; Perez et al., 2023). In addition, down‐regulation of two contiguous genes encoding the 4‐hydroxybenzoate (4HBA) transporter PcaK and a 4HBA monooxygenase (commonly named *p*‐hydroxybenzoate hydroxylase) was also observed (Table 1; Figure S2). 4HBA is a natural intermediate during lignin (and other plant compounds) catabolism and consequently highly abundant in plant environments (An et al., 2023; Chen et al., 2022). In accordance with this genetic potential, we showed that *S. plymuthica* A153 can use PAA, 4HPA and 4HBA as a sole carbon source (Figure S3).

The *hpa* catabolic cluster showed the highest levels of transcriptional changes in our RNA‐seq study (Table 1). This cluster is homologous to the *hpa* gene cluster in *Escherichia coli* strain W (Díaz et al., 2001; Prieto et al., 1996). The *hpaBC* upper operon encodes the enzymes required to convert 4HPA to 3,4‐dihydroxyphenylacetic acid (3,4HPA), whereas the *hpaGEDFHI* meta operon encodes enzymes for the cleavage of the aromatic ring of 3,4HPA (Díaz et al., 2001; Permsirivisarn et al., 2022). It also encodes the AraC/XylS family transcriptional regulator HpaA and the MarR‐type regulator HpaR (Figure S2), which act as an activator and a repressor, respectively, of the upper and meta operons (Díaz et al., 2001; Galan et al., 2003; Prieto & García, 1997). Different genetic and biochemical approaches suggest that 4HPA and 3,4HPA are ligands of HpaR that de‐repress expression of *hpaGEDFHI* (Galan et al., 2003; Permsirivisarn et al., 2022), whereas HpaA may preferentially sense 4HPA (Prieto & García, 1997). However, despite previous attempts (Permsirivisarn et al., 2022), there is, to our knowledge, no information available on the binding parameters of these regulators with their potential cognate ligands. To address this issue, we purified both HpaA and HpaR from A153, which share 62.0% and 66.4% identity, respectively, with homologous proteins in *E. coli*, and conducted isothermal titration calorimetry (ITC) studies. We were unable to observe the binding of HpaR_A153_ to 4HPA or 3,4HPA (Table S4), but demonstrated that HpaA_A153_ bound 4HPA with an affinity of 28.5 μM (Table 2; Figure S4). Given the potential role of IAA in the regulation of the *hpa* cluster, we also investigated whether HpaA_A153_ and HpaR_A153_ bind IAA by conducting additional ITC experiments. Under the conditions tested, we were unable to observe IAA binding by HpaA_A153_ or HpaR_A153_ (Table S4). Furthermore, HpaA_A153_ or HpaR_A153_ did not recognize PAA (Table S4), an auxin that induces the expression of the *hpa* cluster in *E. coli* and that was suggested to be a HpaA ligand (Dierckx et al., 2015; Prieto & García, 1997).

**TABLE 2:** Thermodynamic parameters derived from the microcalorimetric titrations of HpaA and TyrR with different ligands and DNA fragments.

Sample cell ligand	Syringe ligand	*K*_D_ (μM)	Δ*H* (kcal/Mol)
HpaA_A153_	4‐hydroxybenzoic acid	28.5 ± 2	−0.4 ± 0.03
TyrR_A153_	L‐Phe	Binding^a^	
TyrR_A153_	L‐Phe + ATP	193 ± 24	−0.2 ± 0.01
TyrR_A153_	L‐Trp	2681 ± 670	−1.6 ± 0.5
TyrR_A153_	L‐Trp + ATP	*K*_D1_ = 1638 ± 157; *K*_D2_ = 2057 ± 263	Δ*H*_1_ = −1.8 ± 0.3; Δ*H*_2_ = −7.9 ± 1.7
TyrR_A153_	ATP	16.0 ± 0.7	−22.9 ± 1.5
TyrR_A153_	L‐Tyr	No binding	
TyrR_A153_ + ATP	L‐Tyr + ATP	6.0 ± 0.2	−6.8 ± 0.1
TyrR_A153_	Wild‐type DNA^b^	0.193 ± 0.02	7.1 ± 0.1
TyrR_A153_ + L‐Tyr + ATP	Wild‐type DNA^b^ + L‐Tyr + ATP	0.081 ± 0.01	8.2 ± 0.2
TyrR_A153_ + L‐Phe	Wild‐type DNA^b^ + L‐Phe	0.074 ± 0.01	6.3 ± 0.1
TyrR_A153_ + L‐Trp	Wild‐type DNA^b^ + L‐Trp	0.442 ± 0.03	3.3 ± 0.1
TyrR_A153_	Mutant DNA^b^	No binding	

*Note*: Data were analysed using the “One binding site model” of the MicroCal version of ORIGIN. The corresponding data are shown in Figures 6, 7, S4 and S8.

^a^ No satisfactory curve fit was obtained using models in SEDPHAT (Zhao et al., 2015) or the ORIGIN software (MicroCal). The corresponding titration curves are shown in Figure S8.

^b^ 26‐mer DNA fragments of the *ipdc* promoter containing the wild‐type and mutant TyrR box.

### Searching for indole‐3‐acetaldehyde dehydrogenases involved in IAA synthesis in *Serratia plymuthica*

At least five IAA biosynthesis pathways have been described in bacteria that use tryptophan as a precursor (Duca & Glick, 2020). Among them, the IPA pathway is the most commonly found in beneficial phytobacteria (Duca & Glick, 2020; Kunkel & Johnson, 2021). The oxidation step from indole‐3‐acetaldehyde to IAA in the IPA pathway involves an indole‐3‐acetaldehyde dehydrogenase (Duca & Glick, 2020). However, only a limited number of bacterial indole‐3‐acetaldehyde dehydrogenases have been identified (Duca et al., 2014; McClerklin et al., 2018; Shao et al., 2015). In our RNA‐seq analysis, we identified *AWY96_RS13985*, encoding an aldehyde dehydrogenase, as the most down‐regulated gene in the *ipdc* mutant (Table 1). Previously, in an *A. brasilense ipdc* mutant, a repressed gene encoding an aldehyde dehydrogenase was also identified and its role in IAA production hypothesized (Van Puyvelde et al., 2011). To investigate the role of the aldehyde dehydrogenase AWY96_RS13985 in IAA biosynthesis, we generated a deletion mutant in the corresponding gene, but found that this strain produced wild‐type levels of IAA (Figure S5). We subsequently scrutinized the genome of A153 to identify additional candidate indole‐3‐acetaldehyde dehydrogenases. We found that the proteins AWY96_RS19325 and AWY96_RS21200 showed homology with AldH (identity, 71.5%; similarity, 81.2%) and DhaS (identity, 41.6%; similarity, 58.6%) – two aldehyde dehydrogenases that are involved in IAA synthesis in *E. coli* (Guo et al., 2019) and *Bacillus velezensis* (Shao et al., 2015), respectively. To investigate the role of AWY96_RS19325 and AWY96_RS21200 in IAA production in A153, we generated two strains deficient in the corresponding genes. The phenotypic characterization of these strains revealed that both mutants produce wild‐type levels of IAA (Figure S5), suggesting that either there is functional redundancy between aldehyde dehydrogenase enzymes or that A153 encodes an indole‐3‐acetaldehyde dehydrogenase alternative to AWY96_RS13985, AWY96_RS19325 and AWY96_RS21200 yet to be identified.

### TyrR regulates IAA biosynthesis in *S. plymuthica* A153

Our RNA‐seq analyses led to the identification of the gene *AWY96_RS22350*, encoding a transcriptional regulator homologous to the *E. coli* TyrR (TyrR_*Ec*_; Identify, 73.1%), that was down‐regulated in the *ipdc* mutant (Table 1, Figure 4). Given the involvement of TyrR regulators in aromatic amino acid metabolism and transport in other species (Coulson et al., 2020; Patten, 2022; Pittard et al., 2005), we constructed and phenotypically characterized a *tyrR* deletion mutant to investigate the role of TyrR of *S. plymuthica* A153 (TyrR_A153_) in IAA biosynthesis. We found that the *tyrR* mutant was largely impaired in IAA production, showing levels corresponding to ~5% of the wild‐type strain (Figure 2). This phenotype was complemented by the *in trans* expression of *tyrR* in a pBBR‐based plasmid (Figure S6). Consistent with these data, β‐galactosidase assays showed that the deletion of *tyrR* resulted in the abrogation of *ipdc* expression (Figure 1B), indicating the essential role of TyrR_A153_ for IAA synthesis. Subsequent RT‐qPCR analyses confirmed that the transcript levels of *ipdc* were reduced by more than 99% in the *tyrR* mutant (Figure 4B). Due to the involvement of TyrR homologues in the metabolism of additional aromatic compounds (Coulson et al., 2020; Herrera et al., 2010; Patten, 2022; Pittard et al., 2005), we also analysed, by RT‐qPCR, the effect of *tyrR* deletion on the expression of the *paa* and *hpa* catabolic operons in A153. These experiments showed that the *paa* operon was down‐regulated in the *tyrR* mutant, whereas transcript levels of the *hpa* gene cluster remain unchanged (Figure 4B) – indicating that the repression of the *paa* cluster in an *ipdc* mutant was, at least in part, mediated by TyrR_A153_. In accordance with these data, we observed that a *tyrR* mutant showed a lower growth rate compared to the parental strain when grown on a minimal medium with PAA, but not with 4HPA, as a sole carbon source (Figure S3). Further phenotypical characterization of the *tyrR* mutant revealed that this strain exhibits the same antibacterial and antifungal properties as the wild‐type A153 (Figure S7) – phenotypes that are associated with the production of the antimicrobials andrimid (Matilla, Nogellova, et al., 2016) and oocydin A (Matilla et al., 2012), respectively.

### TyrR_A153_ specifically recognizes aromatic amino acids to promote binding to the *ipdc* promoter

**FIGURE 5:**
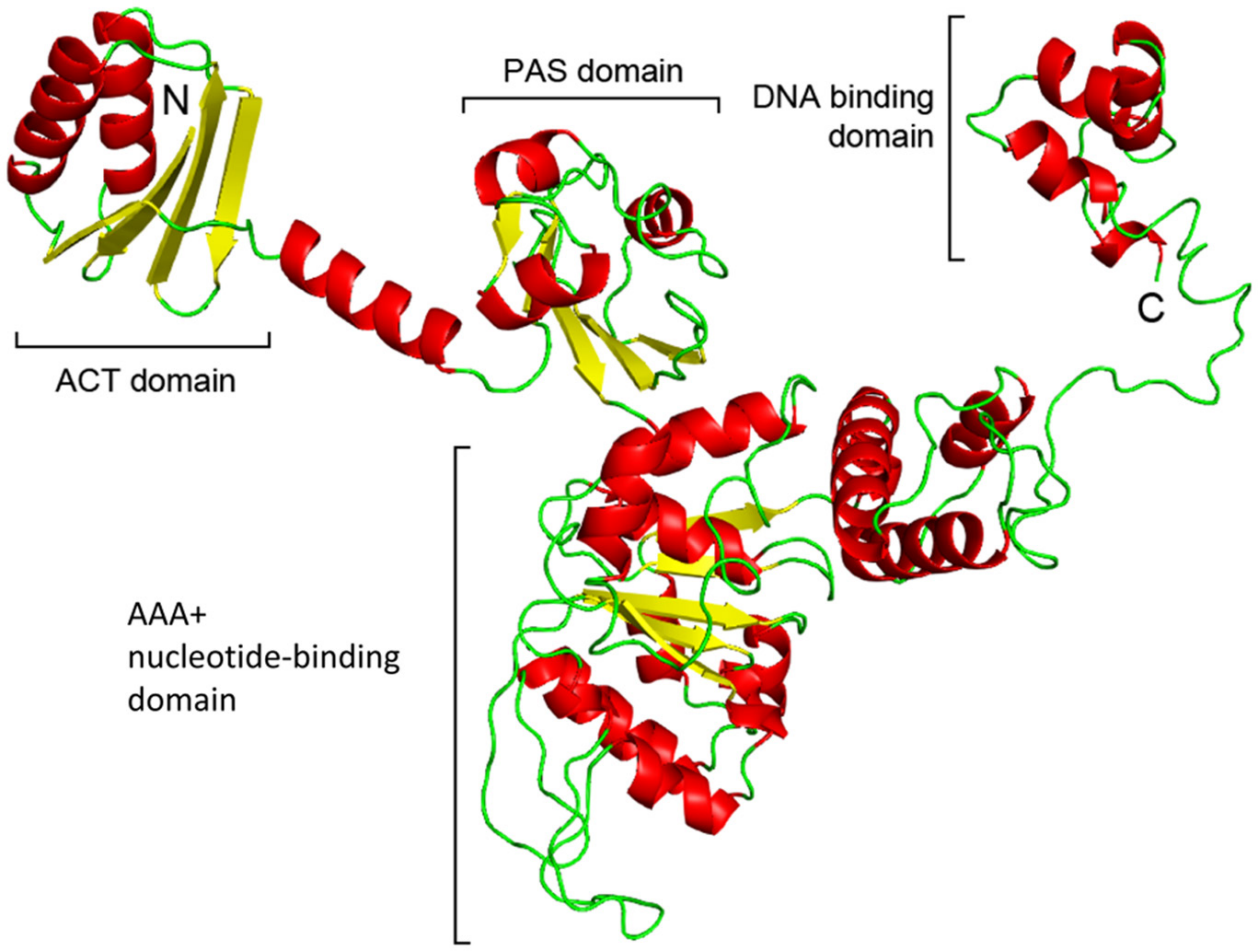
Homology model of TyrR_A153_. The model was generated by Phyre2 (Kelley et al., 2015) using as template structures protein data bank (PDB) entries 2JHE, 1OJL, 1NY5, 3DZD, 6IY8 and 5EP0 (Burley et al., 2019). Ninety‐seven percent of residues were modelled with more than 90% confidence. Protein domain abbreviation: AAA+, ATPase associated with various cellular activities; ACT, Aspartate kinase – chorismate mutase – TyrA; PAS, Per‐Arnt‐Sim.

TyrR_*Ec*_ is an atypical multidomain transcriptional regulator consisting of N‐terminal ACT (for aspartokinase, chorismate mutase, TyrA) and PAS (Per‐Arnt‐Sim) domains, putatively involved in ligand binding and interaction with RNA polymerase, respectively (Pittard et al., 2005). In addition, TyrR_*Ec*_ also contains a central domain with an ATP binding site and an ATP‐dependent ligand binding site, as well as a C‐terminal DNA binding domain (Pittard et al., 2005). TyrR_*Ec*_ was shown to bind each of the three aromatic amino acids to modulate its regulatory capabilities. Whereas L‐Trp and L‐Phe preferentially bound to an ATP‐independent site, L‐Tyr bound to the ATP‐dependent site of TyrR_*Ec*_ (Argaet et al., 1994; Pittard et al., 2005; Wilson et al., 1995). Inspection of a TyrR_A153_ homology model revealed that this protein shares the same domain organization as TyrR_*Ec*_ (Figure 5).

**FIGURE 6:**
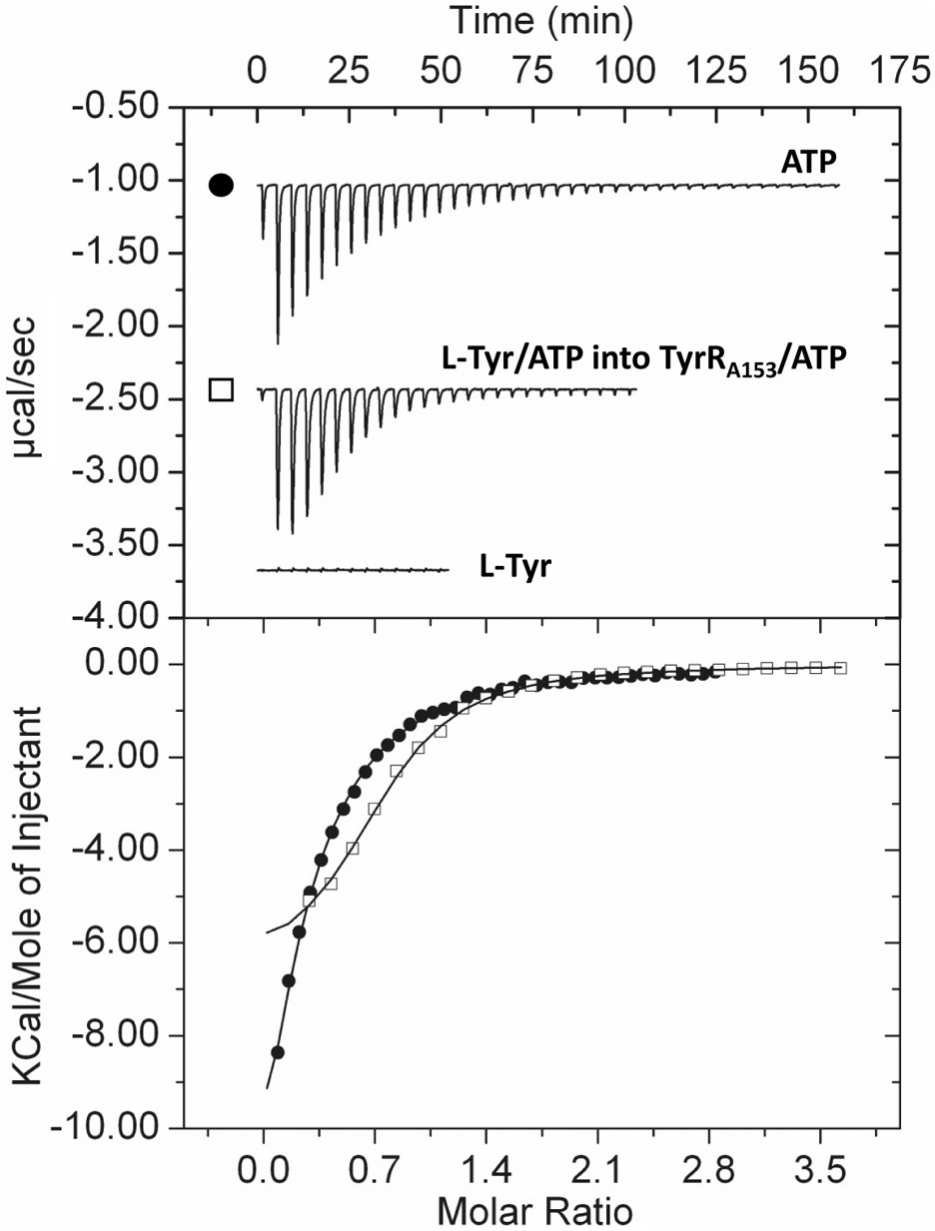
Isothermal titration calorimetry studies of the binding of different ligands to TyrR of *Serratia plymuthica* A153. Upper panel: Raw data for the titration of 50 μM TyrR with 4.8‐ to 12.8‐μL aliquots of 0.5 to 10 mM ligand solutions. Lower panel: Integrated, dilution heat‐corrected and concentration‐normalized peak areas fitted using the ‘One binding site’ model of the MicroCal version of ORIGIN. Thermodynamic parameters are shown in Table 2. Symbols used in the lower panel are defined in the upper panel of this figure. L‐Tyr, L‐Tyrosine.

To study ligand recognition by TyrR_A153_, the protein was purified and binding to amino acids examined by ITC. In the absence of ATP, L‐Phe and L‐Trp bound to TyrR_A153_, whereas no binding of L‐Tyr was observed (Table 2, Figure 6 & Figure S8). Subsequent experiments revealed that TyrR_A153_ bound ATP with a dissociation constant (*K*_D_) of 16 μM (Figure 6, Table 2) in a Mg^2+^‐dependent fashion. In the presence of saturating ATP concentrations, L‐Tyr bound with high affinity (*K*_D_ = 6 μM) (Figure 6, Table 2). To investigate whether the presence of ATP affected L‐Trp and L‐Phe binding to TyrR_A153_, we conducted additional microcalorimetric titrations. TyrR_A153_ bound L‐Phe and L‐Trp with similar affinities in the presence or absence of ATP, although important changes in the binding enthalpies were observed (Table 2 & Figure S8). To investigate whether L‐Phe and L‐Trp competed with L‐Tyr for binding to the ATP‐dependent binding site of TyrR_A153_, we performed competition experiments. In the presence of ATP, no binding of L‐Tyr to TyrR_A153_ was observed in the presence of 10 mM L‐Trp or L‐Phe (Figure S9), revealing that both amino acids compete with L‐Tyr for binding at the ATP‐dependent site of TyrR_A153_. Finally, the ability of TyrR_A153_ to bind additional ligands was analysed by differential scanning fluorimetry‐based ligand screening using commercial libraries comprising ∼450 compounds that serve as bacterial nitrogen, carbon, sulphur or phosphorous sources, as previously described (Fernandez et al., 2018). Unfortunately, no additional ligands were identified, indicating that TyrR_A153_ is a regulator specific for aromatic amino acids.

**FIGURE 7:**
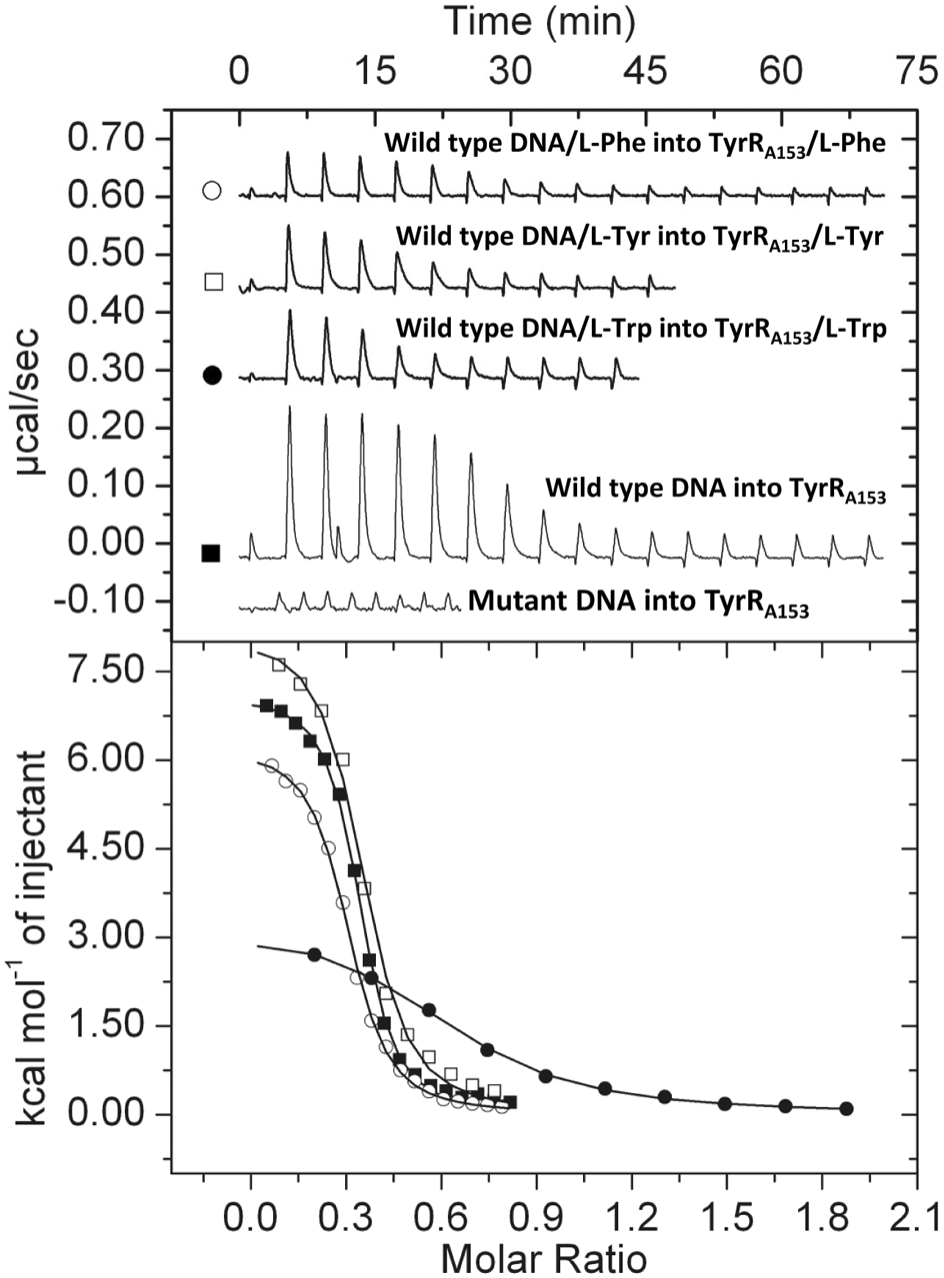
Microcalorimetric titrations of TyrR_A153_ with 26‐bp DNA oligomers of the *ipdc* promoter of *S. plymuthica* A153 containing the wild‐type and mutant TyrR box. Upper panel: Raw data, injection of 3.2–12.8‐μL aliquots of DNA (100 μM) into 5–20 μM TyrR_A153_ in the presence and absence of saturating concentrations of TyrR_A153_ ligands. Lower panel: Integrated, dilution heat‐corrected and concentration‐normalized peak areas fitted using the ‘One binding site’ model of the MicroCal version of ORIGIN. Thermodynamic parameters are shown in Table 2. The symbols used in the lower panel are defined in the upper panel of this figure. L‐Tyr, L‐Tyrosine; L‐Phe, L‐Phenylalanine; L‐Trp, L‐Tryptophan.

We subsequently investigated whether TyrR_A153_ directly regulated the expression of *ipdc*. The analysis of the *ipdc* promoter sequence identified a palindromic sequence TGTAAA‐N_6_‐ATTACA, coinciding with the consensus sequence TGTAAA‐N_6_‐TTTACA recognized by TyrR‐type regulators (Pittard et al., 2005). ITC experiments were conducted to study TyrR_A153_ binding to DNA using a synthetic 26‐bp double‐strand oligomer corresponding to the TyrR box identified within the *ipdc* promoter, flanked by 4 bp extensions on either side. As a negative control, the same double‐strand oligomer containing mutations in the TyrR box was used (Table S3). TyrR_A153_ bound to the wild‐type oligomer in an entropy‐driven process and with very high affinity (*K*_D_ = 193 nM) (Figure 7, Table 2). We found no evidence of binding to the mutant operator (Figure 7). We subsequently evaluated the role of TyrR_A153_ ligands in binding the *ipdc* promoter by ITC. We found that the presence of saturating L‐Tyr and L‐Phe concentrations increased DNA binding affinity 2.4‐ and 2.6‐fold, respectively. In contrast, the presence of L‐Trp reduced the TyrR_A153_ DNA affinity 2.3‐fold (Figure 7, Table 2).

**FIGURE 8:**
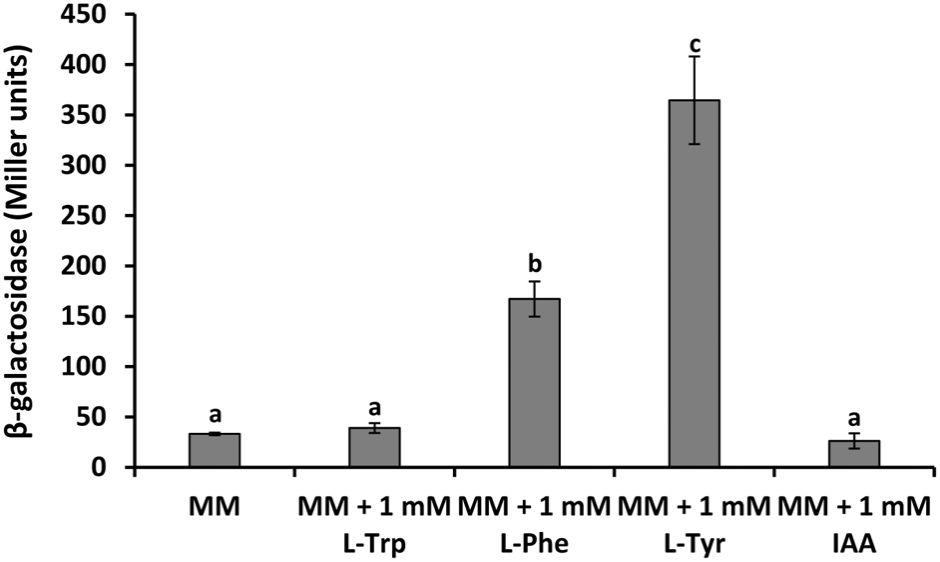
The expression of the *ipdc* gene of *Serratia plymuthica* A153 is induced in the presence of aromatic amino acids. Shown is the transcription of the *ipdc* (P_*ipdc*_ *::lacZ*; pMAMV302) promoter fusion in *S. plymuthica* A153. β‐Galactosidase activity was determined after 24 h of growth in minimal medium (MM) with 15 mM glucose at 30°C in the presence and absence of 1 mM L‐Trp (L‐Tryptophan), L‐Phe (L‐Phenylalanine), L‐Tyr (L‐Tyrosine) or IAA (indole‐3‐acetic acid). Data are the means and standard deviations of three biological replicates. Differences between bars with the same letter are not statistically significant (*p*‐value < 0.01; by Student's *t*‐test).

To investigate the role of L‐Phe, L‐Trp and L‐Tyr on the expression of the *ipdc* gene, transcription from the *ipdc* promoter was assessed in a minimal medium in the absence or presence of 1 mM L‐Trp, L‐Phe or L‐Tyr. In accordance with the ITC data, we found that the expression of the *ipdc* gene was induced 5.1‐ and 11.0‐fold in the presence of L‐Phe and L‐Tyr, respectively, when compared to the same medium lacking these ligands (Figure 8). Conversely, no changes in *ipdc* expression were measured in the presence of L‐Trp. The role of IAA in *ipdc* transcription was also evaluated in minimal medium in the presence of 1 mM of this auxin. No changes in *ipdc* expression were observed (Figure 8). Taken together, these results demonstrate that *ipdc* expression is activated by TyrR_A153_, with L‐Tyr playing a major role in this regulatory process.

### Neither IAA production nor TyrR_A153_ are essential for *S. plymuthica* A153 rhizosphere colonization

An increasing amount of experimental evidence suggests that IAA production by plant‐associated bacteria plays an important role in stablishing interactions between bacteria and plants, including host colonization (Duca et al., 2014; Duca & Glick, 2020; Kunkel & Johnson, 2021; Spaepen & Vanderleyden, 2011). Our transcriptional data show that *ipdc* deletion results in the deregulation of genes potentially involved in rhizosphere colonization and survival (e.g. metabolism of aromatic compounds; stress adaptation; signal transduction; Table 1) (Matilla et al., 2007; Santoyo et al., 2021; Zboralski & Filion, 2020). One of these genes, *flhD*, encodes a flagellar transcriptional regulator (Morgenstein et al., 2010). However, we found no differences in the swimming motility capabilities between the wild‐type strain and the *ipdc* mutant (Figure S10). To examine the role of IAA biosynthesis in rhizosphere colonization by A153, we conducted competitive root colonization assays in maize plants. We found that A153 efficiently colonizes the rhizosphere of maize, with a density of ~7 × 10^8^ bacteria per gram of root. No differences were observed between the competitive root colonizing capacity of the parental strain and that of the *ipdc* mutant (Figure S11A).

TyrR was shown to regulate genes that are important for rhizosphere colonization in different plant‐associated bacteria (Patten, 2022). We investigated the role of TyrR_A153_ in maize rhizosphere colonization in A153 by performing additional root competition assays with the wild‐type strain. These studies showed that a *tyrR*‐deficient strain was not impaired in competitive root colonization (Figure S11B).

### Identification of novel regulators of IAA production in *S. plymuthica* A153

IAA synthesis is tightly regulated in bacteria, both at the transcriptional and post‐transcriptional levels (Zhou et al., 2012; Li et al., 2015; Liu, Wu, et al., 2016; Duca & Glick, 2020). This aspect may be reflected by the observation that (i) no IAA production was detected when A153 was grown in minimal medium (Matilla et al., 2018) and (ii) the presence of the *ipdc* gene in multicopy in A153 results in an IAA overproduction (Figure S6). Given that *ipdc* expression in A153 is growth phase dependent and peaks at stationary phase of growth (Figure 1A), we investigated the role of the stationary phase sigma factor RpoS and quorum sensing (QS) in IAA production. Analysis of the A153 genome (Matilla, Drew, et al., 2016) revealed the presence of a candidate QS locus, named SptIR, as well as two orphan LuxR encoding genes highly homologous to the *splR* and *spsR* genes from the SplIR and SpsIR QS loci from *S. plymuthica* G3 (Liu et al., 2011). We phenotypically characterized *rpoS*, *sptI*, *splR* and *spsR* mutants using the Salkowski reaction as a proxy to determine alterations in IAA levels. We found that the mutation of the acyl homoserine lactone synthase encoding gene *sptI* and *spsR* resulted in an increase IAA production, but no differences were observed for the *splR*‐ and *rpoS*‐deficient strains (Figure S6). Subsequent GC–MS analyses revealed that the *sptI* mutant produces 1.5 times more IAA than the parental strain, whereas a 1.3‐fold increase in IAA production was measured in the *spsR* mutant strain (Figure 2). Our analyses did not reveal a role for SplR and RpoS in IAA production (Figure 2 & Figure S6). We subsequently analysed IAA biosynthesis in a mutant defective in the *pigP* gene, encoding a master transcriptional regulator of secondary metabolism in *Serratia* spp. (Fineran et al., 2005; Shanks et al., 2013). Remarkably, this mutant exhibited a twofold higher IAA production than the wild‐type strain, as measured by GC–MS (Figure 2). Finally, the involvement of the post‐transcriptional regulator Hfq (Kavita et al., 2018) and the non‐coding small RNA of the Csr/Rsm system, *csrB* (Babitzke & Romeo, 2007), in IAA production was analysed. We found no evidence for the involvement of Hfq or *csrB* in IAA synthesis by A153 (Figure 2 & Figure S6).

## DISCUSSION

IAA is an emergent key inter‐ and intra‐kingdom signal molecule in life. Plant‐associated bacteria frequently produce and degrade IAA (Conway et al., 2022; Duca & Glick, 2020; Eichmann et al., 2021; Laird et al., 2020) – activities that result in alterations in the composition of the plant microbiota (Eichmann et al., 2021; Lopes et al., 2023), plant growth promotion (Conway et al., 2022; Eichmann et al., 2021; Spaepen & Vanderleyden, 2011) and alterations in plant virulence (Kunkel & Johnson, 2021). Given the widespread phylogenetic distribution of the genetic potential to synthesize IAA within the bacterial kingdom (Duca et al., 2014; Liu et al., 2021; Zhang et al., 2019), there is growing evidence for this auxin playing an important role as a bacterial signal molecule regulating multiple physiological and metabolic processes (Conway et al., 2022; Duca & Glick, 2020; Gavira et al., 2023; Krell et al., 2023; Kunkel & Johnson, 2021; Rico‐Jiménez et al., 2022). Therefore, it is not surprising that bacterial IAA production is highly regulated by a variety of transcriptional and post‐transcriptional regulators, signal molecules (e.g. quorum sensing signals, 2,4‐diacetylphloroglucinol, plant extracts) and environmental cues (e.g. oxygen, temperature, pH, osmotic stress) in a strain‐dependent manner (Duca & Glick, 2020; Jung et al., 2020; Kunkel & Johnson, 2021). For example, RpoS and Hfq were shown to regulate IAA biosynthesis in various bacteria (Oh et al., 2013; Patten & Glick, 2002b; Saleh & Glick, 2001), including *S. plymuthica* (Liu, Chen, et al., 2016; Liu, Wu, et al., 2016; Zhou et al., 2012). However, despite the fact that IAA production in *S. plymuthica* A153 occurs in the stationary phase, we did not observe an effect of *rpoS* and *hfq* deletion on IAA biosynthesis. Therefore, further research is needed to elucidate the diversity of mechanisms that control bacterial IAA production.

We have established here that different regulatory proteins play an important role in the coordination of IAA production in a biocontrol rhizobacterium. A key regulator in this process is TyrR – a multidomain transcriptional regulator that was initially shown to modulate the biosynthesis and uptake of aromatic amino acids in *E. coli* (Patten, 2022; Pittard et al., 2005). Subsequent work conducted in alternative bacterial models found that TyrR homologues control additional processes, including amino acid catabolism (Bai & Somerville, 1998; Katayama et al., 1999), the glyoxylate shunt (Rodionov et al., 2011), antibiotic production (Coulson et al., 2020; Lango‐Scholey et al., 2013), gluconeogenesis (Coulson et al., 2020), biofilm formation (Jijón‐Moreno et al., 2019), nitrogen assimilation (Deng et al., 2015) and stress response (Coulson et al., 2020). In addition, the expression of the *ipdc* gene was found to be regulated by a TyrR homologue in *Enterobacter ludwigii* (Coulson et al., 2020; Ryu & Patten, 2008), but not in *Azospirillum brasilense* (Jijón‐Moreno et al., 2019). TyrR homologues act either as activators or repressors, sometimes exhibiting both regulatory activities on the same promoter depending on the associated ligand (Patten, 2022; Pittard et al., 2005) – a feature that established TyrR_*Ec*_ as a model protein when studying gene regulation (Patten, 2022; Pittard et al., 2005).

At the beginning of our studies, no data were available on the role of TyrR within the *Serratia* genus. Here, we showed that TyrR_A153_ bound ATP and all three aromatic amino acids in either an ATP‐dependent or independent manner. These observations were consistent with the identification of an ATP‐independent and ATP‐dependent binding site for aromatic amino acids in TyrR_*Ec*_ (Pittard et al., 2005). Both L‐Tyr and L‐Phe, but not L‐Trp, activated *ipdc* expression in A153; with L‐Tyr exhibiting a higher inducer capacity, which was consistent with the higher binding affinity of TyrR_A153_ for this amino acid (Table 2). Given that L‐Phe and L‐Tyr are not known to be involved in IAA metabolism, the biological role of sensing either amino acid in the activation of IAA production in A153 is currently unknown. However, this could be because the IPDC enzyme of A153 can have a broad substrate specificity; as shown previously in other bacterial species (Costelloe et al., 2008; Schütz et al., 2003; Somers et al., 2005). Thus, the regulatory role of L‐Phe and L‐Tyr may be related to the synthesis to additional enzymatic products. In support of this hypothesis, we observed no transcriptional activity of the *ipdc* promoter in an *ipdc*‐deficient mutant (Figure 1A). This transcriptional activity could not be restored by the addition of exogenous IAA (Figure S1), suggesting that an alternative product of the IPDC activity could be acting as an inducer signal of the expression of *ipdc* in A153.

L‐Tyr and L‐Phe sensing caused an increase in the affinity of TyrR_A153_ for binding to the *ipdc* promoter. Previously, TyrR_*Ec*_ was shown to form hexamers in the presence of L‐Tyr and ATP, allowing it to also bind at low affinity sites in target promoters, thereby causing alterations in its regulatory activities (Patten, 2022; Pittard et al., 2005). We hypothesize that a similar mechanism may occur in TyrR_A153_, explaining why higher *ipdc* expression was observed in the presence of L‐Tyr. Contrary to our results, higher *ipdc* expression was observed in the presence of L‐Phe and L‐Trp with respect to L‐Tyr in *E. ludwigii* (Coulson & Patten, 2015) – highlighting the distinct role of these ligands in the regulatory activity of IAA production by TyrR regulators. To our knowledge, this is the first report to determine the ligand binding constants of a TyrR regulator to a promoter of a gene involved in IAA biosynthesis. This knowledge contributes to a broadening profile of regulatory activities and mechanisms by which the TyrR family of transcriptional regulators act. Aromatic amino acids are present in the rhizosphere (Vives‐Peris et al., 2020), suggesting that IAA production is induced in the natural niche of *S. plymuthica* A153.

To our knowledge, there is only a single study that investigates the role of endogenous IAA on the global bacterial transcriptome – a study that was performed by investigating an *ipdc* mutant of *A. brasilense* (Van Puyvelde et al., 2011). In this analysis, the percentage of DEGs was in the same range (~2.0%) as the number of genes with altered expression identified here in *S. plymuthica* (~1.0%). In both studies, DEGs cover functions as diverse as metabolism, stress adaptation, transport and signal transduction – traits known to be important for rhizosphere colonization and survival (Matilla et al., 2007; Santoyo et al., 2021; Zboralski & Filion, 2020). Genes up‐regulated in the wild‐type A153 were clustered around several operons involved in the transport and catabolism of various aromatic compounds. Subsequently, we showed that A153 can efficiently metabolize PAA and 4HBA, which are lignin‐related aromatic compounds (An et al., 2023). These findings support that A153 may be metabolizing aromatic compounds in the rhizosphere and opens the possibility that this strain could be developed as part of biodegradation strategies (An et al., 2023).

The auxin PAA is produced by plants and bacteria from phenylalanine (Patten, 2022), and various pieces of evidence suggest that the metabolic routes for PAA and IAA are the same (Patten et al., 2013; Somers et al., 2005). Therefore, further A153 work exploring the implication of the IPDC enzyme in PAA biosynthesis is required. In this sense, there are parallels between the regulation of IAA biosynthesis and the PAA catabolism in A153, as both activities are positively regulated by the TyrR_A153_. In contrast to our findings, TyrR was shown to repress the *paa* operon in *E. ludwigii* (Coulson et al., 2020), again highlighting the need to analyse TyrR regulatory activities in different model bacteria. Although TyrR_A153_ was not involved in the regulation of the *hpa* operon in A153, other reports suggest that PAA and 4HPA act as effectors of HpaA – a key regulator in 4HPA catabolism (Dierckx et al., 2015; Prieto & García, 1997). However, our ITC data found that HpaA recognized 4HPA but not PAA – stressing the need for conducting further protein‐ligand interaction studies to confirm the identity of signal molecules recognized by sensor proteins (Matilla et al., 2022).

Several important processes during the interaction between plants and bacteria, including biofilm formation, host colonization, motility and chemotaxis, bacterial catabolism and stress resistance are known to be regulated by IAA (Duca & Glick, 2020; Eichmann et al., 2021; Rico‐Jiménez et al., 2022; Spaepen & Vanderleyden, 2011) and TyrR (Patten, 2022). Various genes involved in these activities were differentially expressed in *ipdc* and *tyrR* mutants of A153 (Table 1 and Figure 4), which prompted us to investigate whether either of these mutants was affected in rhizosphere colonization. Under the conditions assayed in this study, we failed to observe a role for auxin production and TyrR‐mediated regulation in rhizosphere colonization, and future work will analyse the transcriptomes of *ipdc*‐ and *tyrR*‐deficient mutants during plant interaction. To our knowledge, there are no studies investigating the role of TyrR in plant colonization, whereas studies with *Pseudomonas putida* showed no role for *ipdc* in the colonization of the rhizosphere (Patten & Glick, 2002a). The production of IAA by *S. plymuthica* A153 and *P. putida* is highly dependent on the presence of L‐Trp (Matilla et al., 2018; Patten & Glick, 2002a). Although the tryptophan content in root exudates can support IAA production by bacteria in the rhizosphere (Kamilova et al., 2006; Liu, Chen, et al., 2016; Liu, Wu, et al., 2016), L‐Trp is present in low concentrations in root exudates (Carvalhais et al., 2015; Lopez‐Farfan et al., 2019). This aspect may be one of the reasons why *ipdc* mutants of *S. plymuthica* and *P. putida* are not affected in their ability to colonize the rhizosphere. In support of this hypothesis, we have previously shown that the phytopathogenic bacterium *Pseudomonas savastanoi* produces high levels of IAA in the absence of L‐Trp (Matilla et al., 2018), and the synthesis of this auxin was critical for *in planta* fitness of this phytopathogen (Aragon et al., 2014). The overexpression of *ipdc* resulted in high IAA levels in A153, indicating that this rhizobacterium has the metabolic potential to synthesize high auxin levels. IAA production is one of the main strategies by which beneficial rhizobacteria promote plant growth (Duca & Glick, 2020; Spaepen & Vanderleyden, 2011) and increasing our understanding of the mechanisms that control IAA synthesis could facilitate the development of novel biotechnological approaches based on microbial phytostimulators.

## CONCLUSIONS

IAA is emerging as a key signal molecule in intra‐ and inter‐kingdom communication. However, there remains a lack of knowledge about the mechanisms by which IAA carries out its regulatory activities and by which its biosynthesis is regulated in microorganisms. In this study, we have shown that deletion of *ipdc* dramatically decreased IAA production in *S. plymuthica* A153 – a rhizospheric biocontrol agent that is a producer of a broad spectrum of antibiotics. The deletion of *ipdc* also resulted in important transcriptomic changes, including altered expression of genes with potential implications for competitive fitness in the rhizosphere. The use of multidisciplinary approaches (e.g. microbial physiology, molecular biology, transcriptomics, analytical chemistry, protein biochemistry or biophysical techniques) has allowed the identification and characterization of different regulators involved in the modulation of IAA production. Given the importance of microbial IAA in plant health and productivity, progress in this field of research is of great relevance given the challenges presented by climate change, increased focus on food security and the requirement for increased crop yields globally.

## AUTHOR CONTRIBUTIONS

**Miriam Rico‐Jimenez:** Data curation (equal); formal analysis (equal); investigation (equal); methodology (equal); writing – review and editing (equal). **Salvador Muñoz‐Mira:** Data curation (equal); formal analysis (equal); investigation (equal); writing – review and editing (equal). **Cristina Lomas‐Martínez:** Investigation (equal); writing – review and editing (equal). **Tino Krell:** Conceptualization (equal); data curation (equal); formal analysis (equal); funding acquisition (equal); methodology (equal); project administration (equal); writing – review and editing (equal). **Miguel A. Matilla:** Conceptualization (equal); data curation (equal); formal analysis (equal); funding acquisition (equal); investigation (equal); methodology (equal); project administration (equal); supervision (equal); writing – original draft (equal); writing – review and editing (equal).

## CONFLICT OF INTEREST STATEMENT

The authors declare that there is no conflict of interest.

## Supporting information


Data S1.

## Data Availability

RNA‐seq data were deposited in the Gene Expression Omnibus repository (accession number GSE226107).
