# Supplementary material for: Regulation of indole‐3‐acetic acid biosynthesis and consequences of auxin production deficiency in Serratia plymuthica
Source: Microb Biotechnol. 2023 Jun 22;16(8):1671–89. doi: 10.1111/1751-7915.14296 (PMC10364317; doi:10.1111/1751-7915.14296)
Supplement: Supplementary file 1 — Data S1. [file MBT2-16-1671-s001.pdf]

Supplementary material to:

Regulation of indole-3-acetic acid biosynthesis and consequences of auxin production deficiency in *Serratia plymuthica*

Miriam Rico-Jiménez[1], Salvador Muñoz-Mira[1], Cristina Lomas-Martínez[1], Tino Krell[1], Miguel A. Matilla[1]*

[1]Department of Biotechnology and Environmental Protection, Estación Experimental del Zaidín, Consejo Superior de Investigaciones Científicas, Prof. Albareda 1, Granada 18008, Spain.

*Address correspondence to Miguel A. Matilla, miguel.matilla@eez.csic.es, Tel. +34 958 526506; Fax +34 958 181609.

Running title: Regulation of auxin production in phytobacteria

Supplementary Tables

Supplementary Table S1. Bacteria, oomycete, fungi, plasmids and oligonucleotides used in this study.

**Bacterial strains**	**Genotype or relevant characteristic**[a]	**Reference or source**
Escherichia coli DH5α	*supE44 lacU169*(*Ø80lacZΔ M15*) *hsdR17* ($r_K^-m_K^-$) *recA1 endA1 gyrA96 thi-1 relA1*	(Woodcock *et al.*, 1989)
E. coli CC118λpir	*araD*, Δ(*ara*, *leu*), Δ*lacZ*74, *pho*A20, *galK*, *thi-1*, *rspE*, *rpoB*, *argE*, *recA1*, λ*pir*	(Herrero *et al.*, 1990)
E. coli BL21(DE3)	F^- *ompT gal dcm lon* $hsdS_B(r_B^-m_B^-)$ λ(DE3 [*lacI lacUV5-T7p07 ind1 sam7 nin5*]) $[malB^+]_{K-12}(\lambda^S)$	(Jeong *et al.*, 2009)
E. coli β2163	F^- RP4-2-Tc::Mu Δ*dapA*::(*erm-pir*); Km^R Em^R	(Demarre *et al.*, 2005)
Serratia plymuthica A153 LacZ	Δ*lacZ* (1470 bp Δ). Wild type strain	(Matilla *et al.*, 2015)
S. plymuthica A153 Δ*ipdc*	A153 Δ*lacZ* , Δ*AWY96_RS14025* (previously *AWY96_14020*)	(Matilla *et al.*, 2018)
S. plymuthica A153 Δ*ipdc*-Km	A153 Δ*lacZ* Δ*ipdc*::Km; Km^R	This study
S. plymuthica A153 Δ*tyrR*-Km	A153 Δ*lacZ* Δ*tyrR*::Km; Km^R	This study
S. plymuthica A153 Δ*AWY96_RS13985*	A153 Δ*lacZ* Δ*AWY96_RS13985*	This study
S. plymuthica A153 Δ*AWY96_RS21200*	A153 Δ*lacZ* Δ*AWY96_RS21200*	This study
S. plymuthica A153 Δ*AWY96_RS19325*	A153 Δ*lacZ* Δ*AWY96_RS19325*::Km; Km^R	This study
S. plymuthica A153 Δ*rpoS*	A153 Δ*lacZ* Δ*rpoS*::Km; Km^R	(Matilla *et al.*, 2015)
S. plymuthica A153 Δ*hfq*	A153 Δ*lacZ* Δ*hfq*::Km; Km^R	(Matilla *et al.*, 2015)
S. plymuthica A153 Δ*sptI*	A153 Δ*sptI*	M.A. Matilla
S. plymuthica A153 Δ*splR*	A153 *splR*::Km; Km^R	M.A. Matilla
S. plymuthica A153 Δ*spsR*	A153 *spsR*::Km; Km^R	M.A. Matilla
S. plymuthica A153 Δ*csrB*	A153 Δ*csrB*	M.A. Matilla
S. plymuthica A153 Δ*pigP*	A153 Δ*lacZ* Δ*pigP*::Km; Km^R	This study
Bacillus subtilis JH642	*pheA1 trpC2*	J.A. Hoch
**Fungi**		
Verticillium dahliae 5368	Wild type, plant pathogen	R. Cooper

[a]Em, erythromycin; Km, kanamycin.

Supplementary Table S2. Plasmids used in this study.

Plasmids	Relevant characteristics[a]	Source
pKNG101	Sm^R; *oriR6K mob sacBR*	(Kaniga *et al.*, 1991)
pUC18Not	Ap^R; identical to pUC18 but with two NotI sites flanking pUC18 polylinker	(Herrero *et al.*, 1990)
pMP220	Tc^R; *oriRK2 ´lacZ*	(Spaink *et al.*, 1987)
p34S-Km3	Ap^R, Km^R; *Km3* antibiotic cassette	(Dennis and Zylstra, 1998)
pBBR1MCS-2_START	Km^R; *oriRK2 mobRK2*	(Obranic *et al.*, 2013)
pBBR1MCS-5_START	Gm^R; *oriRK2 mobRK2*	(Obranic *et al.*, 2013)
pK18mobSacB	Km^R; *oriT mob sacB*	(Schäfer *et al.*, 1994)
pMAMV140	Ap^R; 1.2-kb EcoRI/HindIII PCR product containing *pigP* was inserted into the same sites of pUC18Not	This study
pMAMV146	Ap^R, Km^R; 0.95-kb SmaI fragment containing *km3* cassette of p34S-Km3 was inserted into SmaI site of *pigP* in pMAMV140	This study
pMAMV156	Sm^R, Km^R; 2.2 kb NotI fragment of pMAMV146 was cloned at the same site in pKNG101	This study
pMAMV266	Ap^R; 1.4-kb PCR product containing a 1098 bp in frame deletion of *ipdc* (*AWY96_RS14025*) of A153 inserted into the EcoRI/HindIII sites of pUC18Not	(Matilla *et al.*, 2018)
pMAMV364	Ap^R, Km^R; 0.95 kb BamHI fragment containing *km3* cassette of p34S-Km3 was inserted into BamHI site of *ipdc* in pMAMV266	This study
pMAMV369	Sm^R; Km^R; 2.6-kb NotI fragment of pMAMV364 was cloned at the same site in pKNG101	This study
pMAMV294	Ap^R; 1.5-kb PCR product containing a 1098 bp in frame deletion of *AWY96_RS21200* of A153 inserted into the EcoRI/HindIII sites of pUC18Not	This study
pMAMV297	Sm^R; 1.5-kb NotI fragment of pMAMV294 was cloned at the same site in pKNG101	This study
pUC18-ΔAWY96_RS13985	Ap^R; 1.5-kb PCR product containing a 990 bp deletion of *AWY96_RS13985* of A153 inserted into the EcoRI/HindIII sites of pUC18Not	This study
pK18mobSacB-ΔAWY96_RS13985	Km^R; 1.7-kb EcoRI/HindIII fragment of pUC18-ΔAWY96_RS13985 was cloned at the same sites in pK18mobSacB	This study
pMAMV413	Ap^R; 1.5-kb PCR product containing a 1156 bp deletion of *AWY96_RS19325* of A153 inserted into the EcoRI/HindIII sites of pUC18Not	This study
pMAMV414	Ap^R, Km^R; 0.95 kb BamHI fragment containing *km3* cassette of p34S-Km3 was inserted into BamHI site of *ipdc* in pMAMV413	This study
pMAMV416	Sm^R; Km^R; 2.6-kb NotI fragment of pMAMV414 was cloned at the same site in pKNG101	This study
pMAMV302	Tc^R; *ipdc* promoter region was cloned into the KpnI/SphI sites of pMP220	This study
pUC18tyrR	Ap^R; 2.4-kb BamHI/HindII PCR product containing *tyrR* gene was inserted into the BamHI/HindII sites of pUC18Not	This study

pUC18tyrR-Km3	Ap^R, Km^R; replacement of 0.92-kb EcoRV fragment internal to *tyrR* of pUC18tyrR for a 0.9-kb SmaI *km3* cassette of p34S-Km3	This study
pKNG101-tyrR	Sm^R, Km^R; 3.2 kb NotI fragment of pMAMV53 was cloned at the same site in pKNG101	This study
pET28b-tyrR[b]	Km^R; pET28b(+) derivative containing a DNA fragment encoding the TyrR (AWY96_RS22350) of A153. N-terminal His_6-tag.	This study
pET28b-hpaA[b]	Km^R; pET28b(+) derivative containing a DNA fragment encoding the HpaA (AWY96_RS12560) of A153. N-terminal His_6-tag.	This study
pET28b-hpaR[b]	Km^R; pET28b(+) derivative containing a DNA fragment encoding the HpaR (AWY96_RS12515) of A153. N-terminal His_6-tag.	This study
pBBR-tyrR	Gm^R; NdeI/HindIII fragment from pET28b-tyrR containing the *tyrR* gene was cloned at the same sites in pBBR1MCS-5_START	This study
pBBR-ipdc	Km^R; NdeI/BamHI fragment containing the *ipdc* gene was cloned at the same sites in pBBR1MCS-2_START	This study

[a]Ap, ampicillin; Km, kanamycin; Sm, streptomycin; Tc, tetracycline; Gm, gentamicin.
[b]Constructed by GenScript.

Supplementary Table S3. Oligonucleotides used in this study.

Name	Sequence (5´- 3')	Description	Source
Ipdc-KpnI-F	TAATGGTACCCATCATAAAGCTCCTGTAAAATTAAGGC	Forward primer to clone promoter of *ipdc* into pMP220	This study
Ipdc-SphI-R	TAATGCATGCCATGTGTTTCTCCGGATTTTATCTC	Reverse primer to clone promoter of *ipdc* into pMP220	This study
PigP-EcoRI	GATTGGAATTCCCTGGTGCCAGT	Forward primer to generate a *pigP* mutant of A153	This study
PigP-HindIII	TAATAAGCTTGCAACTCATGGCGGC	Reverse primer to generate a *pigP* mutant of A153	This study
AWY96_RS13985-EcoRI-F	TAATGAATTCGACGCTGAGCCTGACCAACC	Forward primer to clone upstream flanking region of *AWY96_RS13985* for deletion in A153	This study
AWY96_RS13985-PstI-R	TAATCTGCAGCAGATCGGCATAGCGCAGC	Reverse primer to clone upstream flanking region of *AWY96_RS13985* for deletion in A153	This study
AWY96_RS13985-PstI-F	TAATCTGCAGGCCAACGACACCGACTACG	Forward primer to clone downstream flanking region of *AWY96_RS13985* for deletion in A153	This study
AWY96_RS13985-HindIII-R	TAATAAGCTTCCTTACTGATGCCGCGCAG	Reverse primer to clone downstream flanking region of *AWY96_RS13985* for deletion in A153	This study
AWY96_RS21200-EcoRI-F	TAATGAATTCGCTCAGCCCGTAGTGTTGGC	Forward primer to clone upstream flanking region of *AWY96_RS21200* for in-frame deletion in A153	This study
AWY96_RS21200-BamHI-R	TAATGGATCCGATGGCCAGCATCACGTTGG	Reverse primer to clone upstream flanking region of *AWY96_RS21200* for in-frame deletion in A153	This study
AWY96_RS21200-BamHI-F	TAATGGATCCGTCTGGACCAACTGTTATCACCTC	Forward primer to clone downstream flanking region of *AWY96_RS21200* for in-frame deletion in A153	This study
AWY96_RS21200-HindIII-R	TAATAAGCTTGGTGGCGTATTGGATCGCCAG	Reverse primer to clone dowstream flanking region of *AWY96_RS21200* for in-frame deletion in A153	This study
AWY96_RS19325-EcoRI-F	TAATGAATTCGAACAGTGAGGAGTACGCCC	Forward primer to clone upstream flanking region of *AWY96_RS19325* for in-frame deletion in A153	This study
AWY96_RS19325-BamHI-R	TAATGGATCCCAGTCGGTTCTCAATGTTCAGG	Reverse primer to clone upstream flanking region of *AWY96_RS19325* for in-frame deletion in A153	This study
AWY96_RS19325-BamHI-F	TAATGGATCCCGGTGACGTCCTTTAACGGC	Forward primer to clone downstream flanking region of *AWY96_RS19325* for in-frame deletion in A153	This study
AWY96_RS19325-HindIII-R	TAATAAGCTTCAACTCCAGTTGGGTATTGCC	Reverse primer to clone dowstream flanking region of *AWY96_RS19325* for in-frame deletion in A153	This study
TyrR-BamHI-F	TAATGGATCCCTGCATCAATAGCCACCGCAC	Forward primer for *tyrR* mutation	This study

TyrR-HindIII-R	TAATAAGCTTGCAGGCCATCTGCCACAGTC	Reverse primer for *tyrR* mutation	This study
ipdc-NdeI-F	TAATCATATGAAAATCACTATTGGAGCCTTTATTCTG	Forward primer to complement *ipdc* mutation	This study
ipdc-BamHI-F	TAATGGATCCCTGAGAAGCGGCGGATAAC	Reverse primer to complement *ipdc* mutation	This study
ipdc-qPCR-F	CAATTAGTGATCGAGGTTGCCC	Forward primer for qRT-PCR. *ipdc* gene.	This study
ipdc-qPCR-R	CGCCATGTGATCATACAACTCG	Reverse primer for qRT-PCR. *ipdc* gene.	This study
RS13985-F	CGCTGATGATCGGCATGTGG	Forward primer for qRT-PCR. Aldehyde dehydrogenase family protein encoding gene.	This study
RS13985-R	CCAGTCACCACGTTGAATACGC	Reverse primer for qRT-PCR. Aldehyde dehydrogenase family protein encoding gene.	This study
RS12565-F	GGCGCTGACCCACTACAAC	Forward primer for qRT-PCR. *hpaB* gene	This study
RS12565-R	CCACCAGCTCATAGGAGGCG	Reverse primer for qRT-PCR. *hpaB* gene	This study
RS12535-F	CGCGAGCCGTGGAATACATC	Forward primer for qRT-PCR. *hpaD* gene	This study
RS12535-R	CGGTCATCGCGGTATCATGC	Reverse primer for qRT-PCR. *hpaD* gene	This study
RS12515-F	GCATGGAGCGCGATAAGCTG	Forward primer for qRT-PCR. *hpaR* gene	This study
RS12515-R	GCCACGTCGTACAACTCCTG	Reverse primer for qRT-PCR. *hpaR* gene	This study
RS12560-F	CAGCAATCTGGCGCTCGATATG	Forward primer for qRT-PCR. *hpaA* gene	This study
RS12560-R	CCAGTTGCTGGCCGAATTCAC	Reverse primer for qRT-PCR. *hpaA* gene	This study
RS22350-F	GCGCGGTAGTCATGCTCAAG	Forward primer for qRT-PCR. *tyrR* gene	This study
RS22350-R	GGCTGACGGCCACGATATG	Reverse primer for qRT-PCR. *tyrR* gene	This study
RS12485-F	GACATGCGCGATATGACGGAG	Forward primer for qRT-PCR. LuxR encoding gene *AWY96_RS12485*	This study
RS12485-R	CGCGTCGCCAGTTCTATCAG	Reverse primer for qRT-PCR. LuxR encoding gene *AWY96_RS12485*	This study
RS24485-F	CGCAGCCATCGTCAATCAGG	Forward primer for qRT-PCR. *paaA* gene	This study
RS24485-R	CGTTAGCCATCGCCATCACC	Reverse primer for qRT-PCR. *paaA* gene	This study
Pipdc-wt-ITC-F	CCCCTGTAAAGGAGCCATTACATCAA	Forward primer to conduct TyrR-DNA binding studies. Wild type sequence	This study
Pipdc-wt-ITC-R	TTGATGTAATGGCTCCTTTACAGGGG	Reverse primer to conduct TyrR-DNA binding studies. Wild type sequence	This study
Pipdc-mut-ITC-F	TTGACACGGCGGCTCCCACGTCGGGG	Forward primer to conduct TyrR-DNA binding studies. Mutant sequence	This study
Pipdc-mut-ITC-F	CCCCGACGTGGGAGCCGCCGTGTCAA	Reverse primer to conduct TyrR-DNA binding studies. Mutant sequence	This study

Supplementary Table S4. List of compounds that did not bind to HpaA$_{A153}$, HpaR$_{A153}$ and TyrR$_{A153}$ as determined by isothermal titration calorimetry.

Protein	Ligand
**HpaA$_{A153}$**	Phenylacetic acid
	3,4-dihydroxyphenylacetic acid
	3-methylbenzoic acid
	Benzoic acid
	Salicylic acid
	Indole-3-acetic acid
	Indole-3-pyruvic acid
**HpaR$_{A153}$**	4-hydroxybenzoic acid
	3-hydroxyphenylacetic acid
	Phenylacetic acid
	3,4-dihydroxyphenylacetic acid
	3-methylbenzoic acid
	Benzoic acid
	Salicylic acid
	Indole-3-acetic acid
	Indole-3-pyruvic acid
**TyrR$_{A153}$**	Indole-3-acetic acid

Supplementary Figures

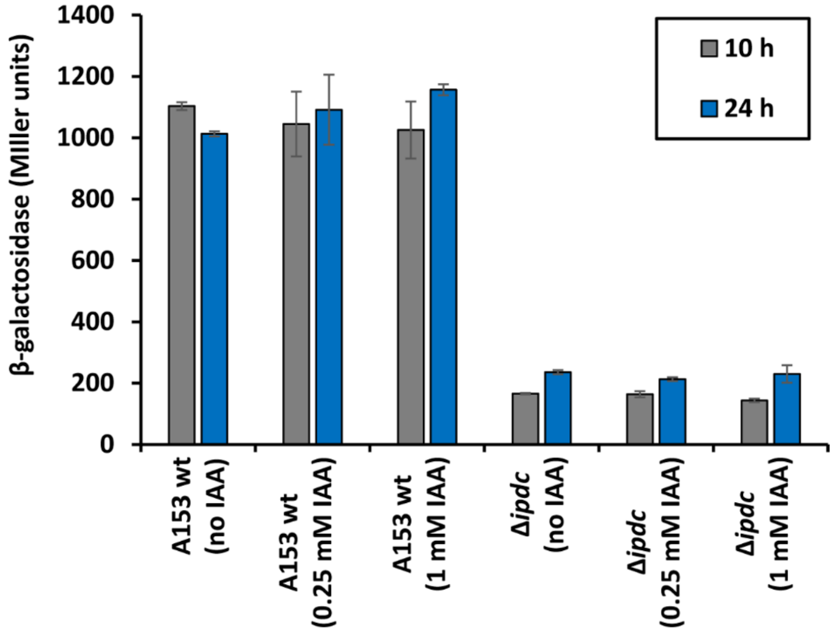

Fig. S1. The expression of the *ipdc* gene of *Serratia plymuthica* A153 is not affected by the exogenous addition of indole-3-acetic acid (IAA). Transcription of the *ipdc* (P_{ipdc}::*lacZ*; pMAMV302) promoter in LB medium at 30 °C in the absence and presence of different concentrations of IAA. Data are the mean and standard deviation of three biological replicates. No significant differences were found in the presence and absence of exogenous IAA. Wt, wild-type.

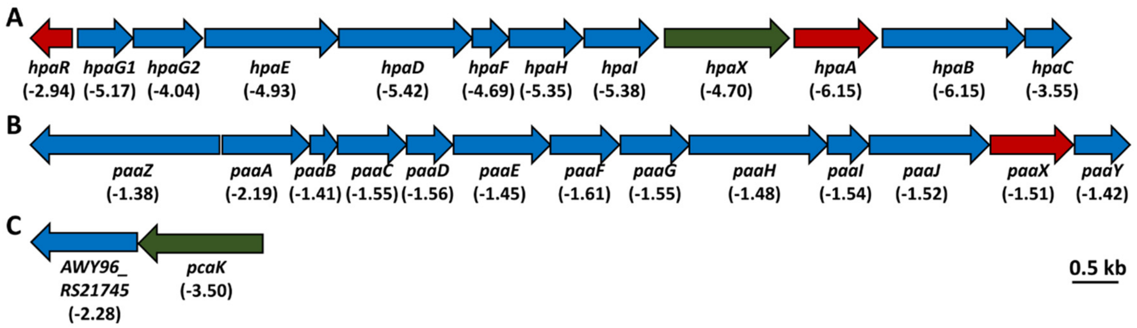

Fig. S2: Genetic organization of the 4-hydroxyphenylacetic acid (A), phenylacetic acid (B) and 4–hydroxybenzoate (C) catabolic gene clusters in *Serratia plymuthica* A153. Numbers in brackets correspond to the results of the RNA-seq studies (*ipdc* mutant versus A153 wild type). Green: transporter/permease; red, regulatory protein; blue: catabolic genes.

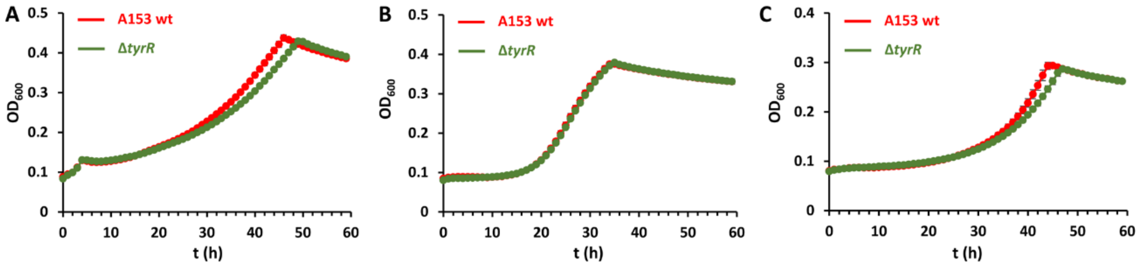

Fig. S3. Growth curves of *Serratia plymuthica* A153 strains in minimal medium with phenylacetic acid (PAA), 4-hydroxyphenylacetic acid (4HPA) and 4-hydroxybenzoic acid (4HBA) as sole carbon sources. Growth experiments were conducted in 100-well plates in minimal medium supplemented with 5 mM of PAA (**A**), 4HPA (**B**) and 4HBA (**C**). Data represent growth for 60 h. Cells were grown at 30 °C using a Bioscreen microbiological growth analyzer (Oy Growth Curves Ab Ltd., Helsinki, Finland) under continuous shaking. Wt, wild-type; OD_{600}, optical density at 600 nm.

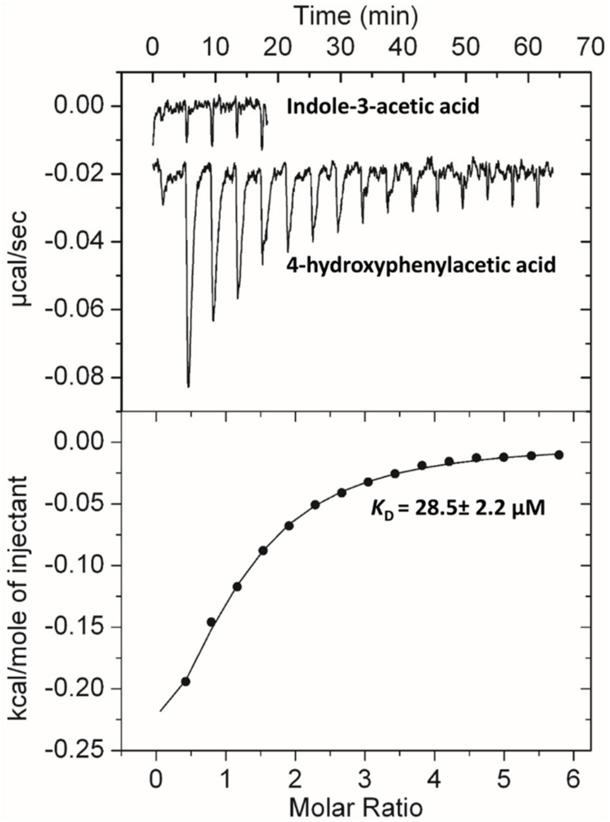

Fig. S4. Isothermal titration calorimetry study of the binding of different ligands to HpaA of *Serratia plymuthica* A153. Upper panel: Raw data for the titration of 22 to 36 µM HpaA with 8- to 9.6-µL aliquots of 2 to 3 mM ligand solutions. Lower panel: Integrated, dilution heat-corrected and concentration-normalized peak areas fitted using 'One binding site' of the MicroCal version of ORIGIN. Thermodynamic parameters are shown in Table 2.

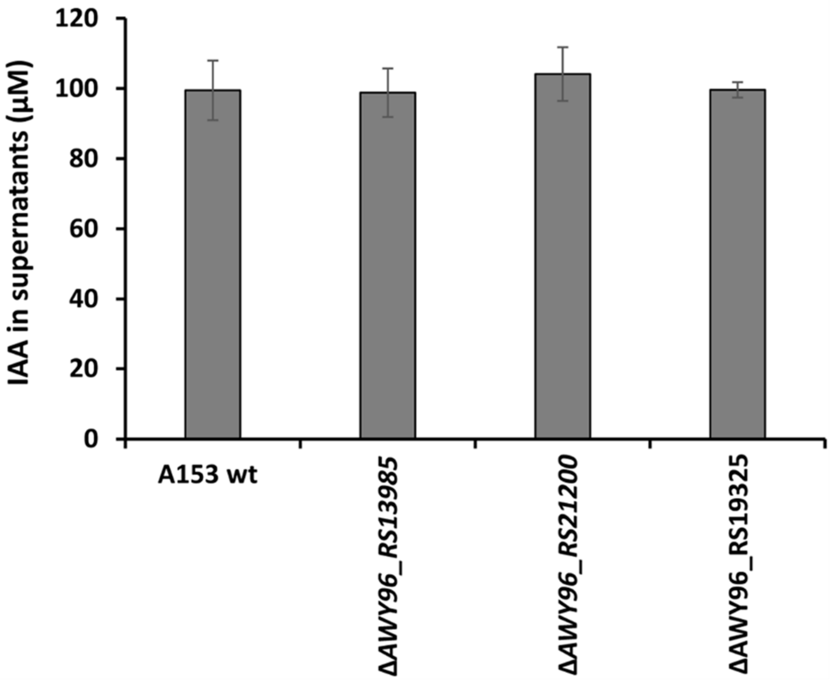

Fig. S5. Role of different aldehyde dehydrogenases in IAA production in *S. plymuthica* A153. Assays were performed in LB broth in the presence of 1 mg/mL L-Trp. Means and standard deviations of three biological replicates are shown. Samples were taken after 24 h incubations at 30 ºC. No significant differences were found in the levels of IAA between A153 strains. Wt, wild-type.

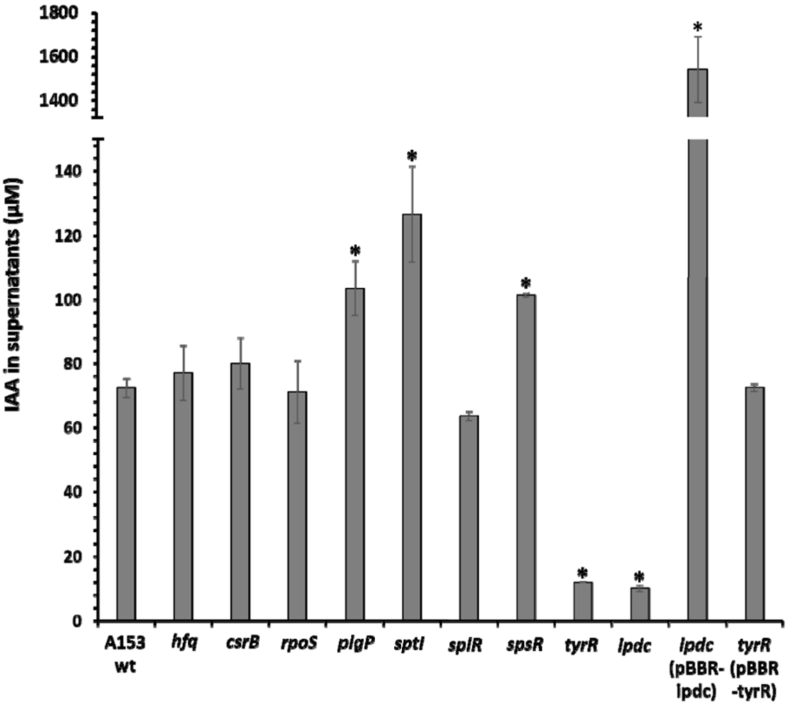

Fig. S6. Indole-3-acetic acid production by different *Serratia plymuthica* A153 strains. Assays were performed in LB broth in the presence of 1 mg/mL L-tryptophan. Means and standard deviations of three biological replicates are shown. Samples were taken after 24 h incubations at 30 ºC. *P < 0.01, Student's t-test of mutant strains with respect to the A153 wild-type strain. Wt, wild-type.

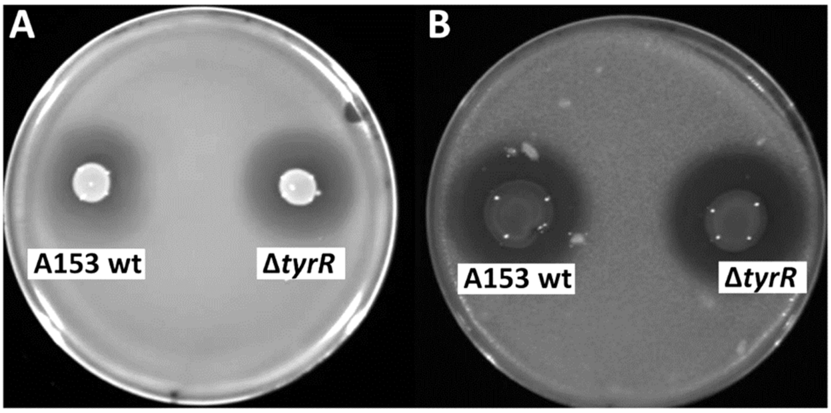

Figure S7. Antibacterial and antifungal properties of *Serratia plymuthica* A153 strains. Shown are the antimicrobial activities against *Bacillus subtilis* (A) and *Verticillium dahliae* (B) after 24 h and 96 h of growth at 25 ºC, respectively.

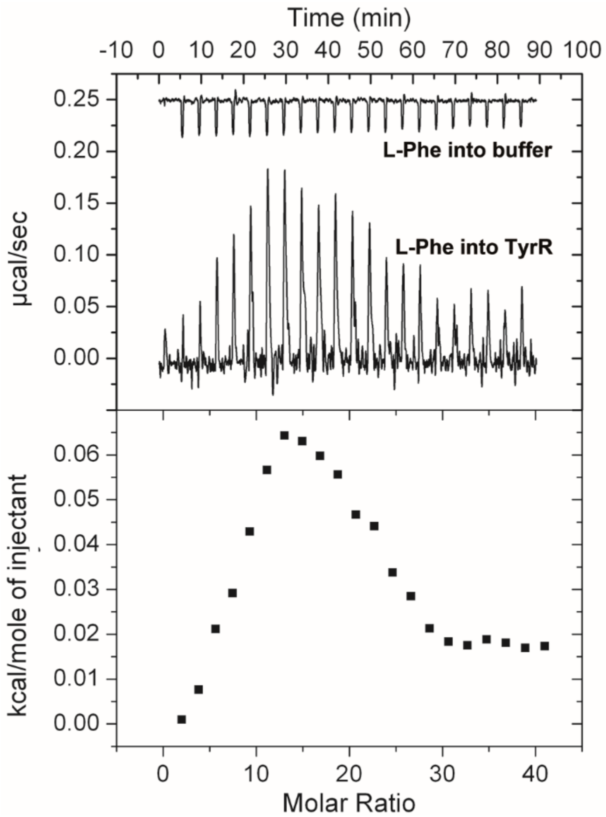

Figure S8. Isothermal titration calorimetry study of the binding of L-Phe to TyrR$_{A153}$. Upper panel: Raw data for the titration of 50 μM TyrR$_{A153}$ with 12.8-μL aliquots of 10 mM L-Phe. Lower panel: Integrated, dilution heat-corrected and concentration-normalized peak areas. No satisfactory fit was obtained with models in the SEDPHAT (Zhao *et al.*, 2015) or the ORIGIN software (MicroCal). L-Phe, L-Phenylalanine.

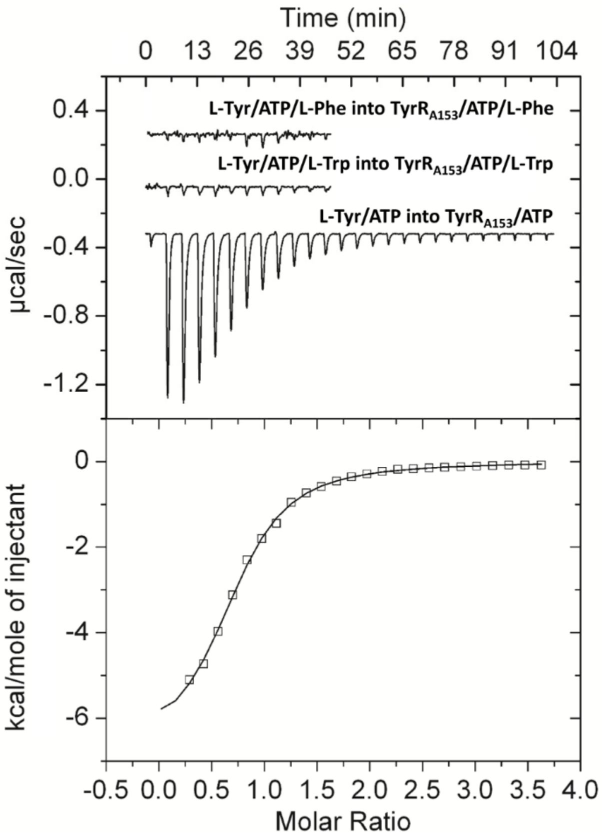

Figure S9. L-Phe and L-Trp compete with L-Tyr for binding to the ATP-dependent binding site of TyrR$_{A153}$. Shown are the results from isothermal titration calorimetry analysis of the binding of L-Tyr to the ATP-dependent binding site of TyrR$_{A153}$ in the presence and absence of 10 mM L-Phe or L-Trp. Upper panel: titration raw data for the injection of 4.8-12.8 µL aliquots of 1 mM L-Tyr into 50 µM TyrR$_{A153}$ in the absence and presence of 10 mM L-Phe or L-Trp (present in both the injector syringe and sample cell). Lower panel: integrated, dilution heat-corrected and concentration-normalized peak areas fitted with the “One binding site” model of ORIGIN. In all cases, 1 mM ATP was present both in the injector syringe and sample cell. L-Phe, L-Phenylalanine; L-Trp, L-Tryptophan; L-Phe, L-Phenylalanine.

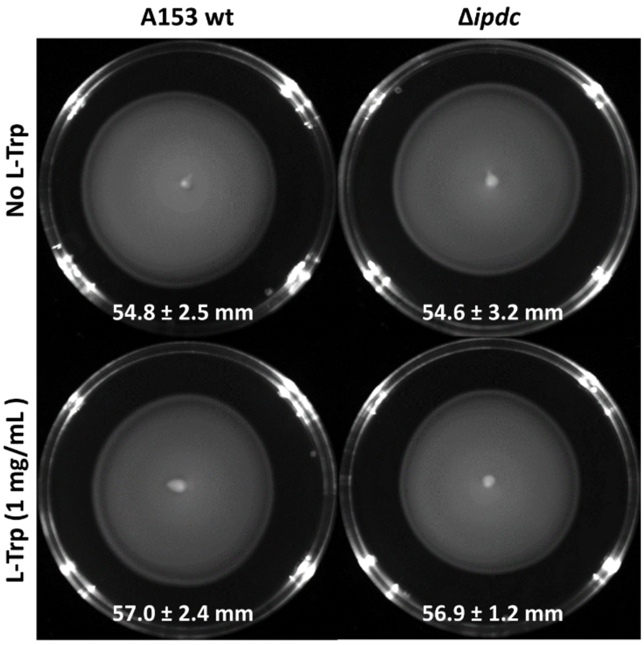

Fig. S10. Swimming motility after of *Serratia plymuthica* A153 strains in the presence and absence of 1 mg/mL L-tryptophan. Numerical values at the bottom of each bioassay represent the mean and standard deviation of halo diameters from three biological replicates. Each of these assays was conducted three times and representative images are shown. No significant differences in swimming motility were found between A153 strains. Pictures were taken after 24 h of incubation at 30 ºC. L-Trp, L-tryptophan; wt, wild-type.

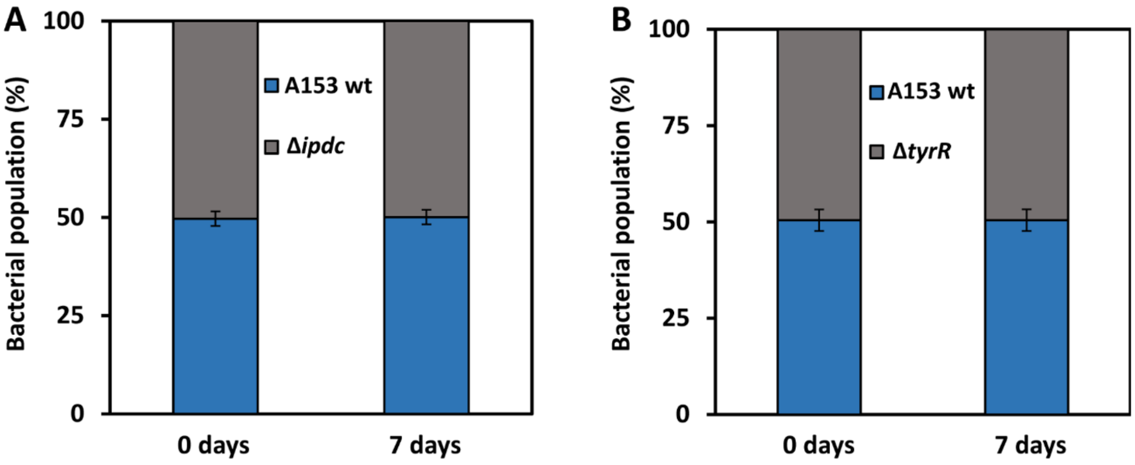

Fig. S11. Competitive root colonization of *Serratia plymuthica* A153 and mutants defective in *ipdc* (A) and *tyrR* (B). The figures present the percentage of bacteria recovered from the rhizosphere of maize (*Zea mays*) plants. Data are the means and standard deviations of six plants. No significant differences in the rhizosphere colonization levels were found between A153 strains. Wt, wild-type.

REFERENCES

Demarre, G., Guerout, A.M., Matsumoto-Mashimo, C., Rowe-Magnus, D.A., Marliere, P., and Mazel, D. (2005) A new family of mobilizable suicide plasmids based on broad host range R388 plasmid (IncW) and RP4 plasmid (IncPalpha) conjugative machineries and their cognate *Escherichia coli* host strains. *Res Microbiol* **156**: 245–255.

Dennis, J.J. and Zylstra, G.J. (1998) Plasposons: modular self-cloning minitransposon derivatives for rapid genetic analysis of gram-negative bacterial genomes. *Appl Environ Microbiol* **64**: 2710–2715.

Herrero, M., de Lorenzo, V., and Timmis, K.N. (1990) Transposon vectors containing non-antibiotic resistance selection markers for cloning and stable chromosomal insertion of foreign genes in gram-negative bacteria. *J Bacteriol* **172**: 6557–6567.

Jeong, H., Barbe, V., Lee, C.H., Vallenet, D., Yu, D.S., Choi, S.H., et al. (2009) Genome sequences of *Escherichia coli* B strains REL606 and BL21(DE3). *J Mol Biol* **394**: 644–652.

Kaniga, K., Delor, I., and Cornelis, G.R. (1991) A wide-host-range suicide vector for improving reverse genetics in Gram-negative bacteria: inactivation of the *blaA* gene of *Yersinia enterocolitica*. *Gene* **109**: 137–141.

Matilla, M.A., Daddaoua, A., Chini, A., Morel, B., and Krell, T. (2018) An auxin controls bacterial antibiotics production. *Nucleic Acids Res* **46**: 11229–11238.

Matilla, M.A., Leeper, F.J., and Salmond, G.P. (2015) Biosynthesis of the antifungal haterumalide, oocydin A, in *Serratia*, and its regulation by quorum sensing, RpoS and Hfq. *Environ Microbiol* **17**: 2993–3008.

Obranic, S., Babic, F., and Maravic-Vlahovicek, G. (2013) Improvement of pBBR1MCS plasmids, a very useful series of broad-host-range cloning vectors. *Plasmid* **70**: 263–267.

Schäfer, A., Tauch, A., Jäger, W., Kalinowski, J., Thierbach, G., and Pühler, A. (1994) Small mobilizable multi-purpose cloning vectors derived from the *Escherichia coli*

plasmids pK18 and pK19: selection of defined deletions in the chromosome of *Corynebacterium glutamicum*. *Gene* **145**: 69–73.

Spaink, H.P., Okker, R.J., Wijffelman, C.A., Pees, E., and Lugtenberg, B.J. (1987) Promoters in the nodulation region of the *Rhizobium leguminosarum* Sym plasmid pRL1JI. *Plant Mol Biol* **9**: 27–39.

Woodcock, D.M., Crowther, P.J., Doherty, J., Jefferson, S., DeCruz, E., Noyer-Weidner, M., et al. (1989) Quantitative evaluation of *Escherichia coli* host strains for tolerance to cytosine methylation in plasmid and phage recombinants. *Nucleic Acids Res* **17**: 3469–3478.
